# Effects of the COVID-19 pandemic on hospice and palliative care in nursing homes—A qualitative study from a multiperspective view

**DOI:** 10.1371/journal.pone.0286875

**Published:** 2023-10-05

**Authors:** Anna Bußmann, Natalie Pomorin, Vera Gerling, Hendrik Wolthaus, Anne-Katrin Teichmüller

**Affiliations:** 1 Essener Forschungsinstitut für Medizinmanagement GmbH, Essen, North Rhine-Westphalia, Germany; 2 FOM Hochschule für Oekonomie & Management Gemeinnützige Gesellschaft mbH, Düsseldorf, North Rhine-Westphalia, Germany; 3 Forschungsgesellschaft für Gerontologie e.V. Institut für Gerontologie an der TU Dortmund, Dortmund, North Rhine-Westphalia, Germany; 4 Contilia Pflege und Betreuung GmbH, Essen, North Rhine-Westphalia, Germany; World Health Organization, BELGIUM

## Abstract

In Germany, nursing homes are characterised by challenging conditions for adequately supporting residents at their end of life, which have even amplified due to the COVID-19 pandemic. This article therefore analyses how hospice and palliative care in nursing homes has changed due to the COVID-19 pandemic and how the pandemic has affected residents, relatives and employees. Semi-structured interviews with employees, residents and relatives were conducted before and during the COVID-19 pandemic in two nursing homes in North Rhine-Westphalia, Germany. In a pre-post comparison, data were qualitatively evaluated using content analysis according to Mayring. Shifts, congruities and discrepancies in challenges in hospice and palliative care were identified between T0 and T1. Due to contact restrictions, important parts of end-of-life care were missing, and the roles of individuals providing hospice and palliative care were redefined. The interviewed groups experienced changes differently and contradictory statements on satisfaction and expectations about hospice and palliative care were reported. Employees and relatives predominantly perceived the pandemic to be very stressful, while residents endured this period more composedly. Employees stated that, despite the pandemic, they were mostly able to meet residents’ requests. However, relatives and residents expressed that minor requests were not reliably fulfilled, neither at T0 nor at T1. Drawing together the different perspectives from employees, residents and relatives offers a bigger picture of challenges in hospice and palliative care in nursing homes and the pandemic effects. Stronger communication of requests and needs as well as greater collaboration, especially under crisis conditions, are essential for a better quality of end-of-life care. There is an urgent need to break down the taboos around the topics of dying and death in nursing homes.

## Introduction

In Germany, nursing homes are second only to hospitals as the place in which the greatest number of people’s lives end [1]. Currently, residents enter these facilities later, stay for shorter periods and, in most cases, have multiple diseases and cognitive functional impairments [2, 3]. Therefore, palliative and hospice care for people in the last phase of life is becoming increasingly important in nursing homes [1, 4–6].

Palliative care is focused on quality of life and individual needs based on a holistic consideration of social, spiritual, physical and psychological forms of pain and suffering [7–9]. A palliative care team includes multiple different professionals, such as specialised doctors and nurses, social workers, therapists and chaplains [10]. These professions work with patients and their families to provide medical, social, emotional, and practical support at home, in hospitals, in nursing homes or in other facilities [10].

Hospice care arose out of a citizen movement that wanted to ensure that no one had to die alone. It is primarily carried out by volunteers and can be offered at home or in different facilities, e.g., nursing homes [10]. Hospice care focuses on the quality of life and comfort of a person in the last phase of life and on their family [10].

German national recommendations for palliative care emphasise that particular attention should be given to the needs of residents and their relatives [11]. At the same time, nursing homes are characterised by challenging structural, organisational and staffing conditions. The framework and working conditions in nursing homes have led to increasing workloads, high quantitative work requirements as well as time and performance pressure [12, 13], which make it difficult to adequately support residents at the end of their lives and focus on their needs [14]. Various studies demonstrate that transferring existing concepts of hospice and palliative care into practice has only been marginally successful and collaborations, for example with outpatient hospice services, are not yet common practice [9, 15].

Despite a higher level of required care, general palliative care counts as part of nursing care in Germany and is not specifically considered in the financing of nursing homes [8, 16, 17]. As a result, care facilities are caught between the ethical demand for appropriate end-of-life care and limited financial and human resources [18].

The start of the COVID-19 pandemic and the first cases in Germany at the end of January 2020 posed new challenges for nursing homes. First, nursing home residents are a highly vulnerable group that is at particular risk of suffering from severe progression of COVID-19 and death as a consequence [19]. Second, infections very quickly spread in shared accommodations and outbreaks are common [20]. The result is that nursing homes in Germany were significantly impacted by the effects of the pandemic [20].

The greater likelihood of infection together with the increased probability of a severe or fatal course of the disease has brought end-of-life care into even greater focus [21]. At the same time, protective measures against COVID-19 taken in March 2020 made it virtually impossible to provide personalised support and care based on individual needs for high-quality end-of-life care. These protective measures included contact restrictions, quarantine regulations, the suspension of group activities and greater pressure on human resources due to the fulfilment of hygiene requirements together with staff shortages as a result of the pandemic [20].

As a consequence, employees at German nursing homes have reported a significant rise in the physical and mental workload [22–24]. Hygiene measures and changing information on infection prevention require the daily adjustment of workflows in nursing homes, which prevents routine processes and heightens emotional stress [22]. Increased staff shortages due to quarantine measures, exacerbated by the absence of volunteers as well as the visiting restrictions for relatives, who otherwise assist with end-of-life care and support, add to the time pressure [20, 25]. In addition, employees are mentally burdened by their own risk of infection as well as the fear of infecting residents and their own social circle [23]. Consequently, employees at nursing homes have described a conflict between adequate palliative care in terms of personal, psychosocial and emotional support and compliance with hygiene measures, the result of which is that they cannot provide residents with their customary care [22].

At the time of this research, very few studies exist on the effects of the coronavirus pandemic on residents and relatives that survey these target groups themselves. An investigation by Kaelen et al. (2021) demonstrated that residents in Belgian nursing homes experienced a loss of freedom, their social life, their autonomy and their leisure activities, which deprives residents of their basic needs. According to the study, this loss has a massive impact on their mental well-being, expressed in feelings of depression, anxiety, and frustration as well as decreased meaning and quality of life [26]. In contrast, an investigation by Schweighart et al. (2021) demonstrated that residents with depressive symptoms in a German nursing home had very different experiences of the pandemic and that the effects are wide-ranging. In particular, people who indicated that they had lived their life and are ready to die reported no additional mental stress because of COVID-19 or the fear of a possible infection. Other respondents were more stressed by their concern for relatives, loneliness and generally increased anxiety [27]. In a survey by Wammes et al. 2020, relatives of residents of Dutch nursing homes particularly reported increased loneliness (73%) and fear (26%) [28].

Although there are few studies about pandemic effects on palliative care in Germany [29–35] as well as pandemic effects on nursing homes in Germany [20, 24, 36–40], at the time of article publication, no studies could be found on hospice and palliative care in German nursing homes under pandemic conditions. In particular, there are no studies in which the different perspectives of employees, residents and their relatives are combined. There are also no studies that analyse the changes and effects of the COVID-19 pandemic from pre-pandemic perspectives of the same sample. This article therefore examines the extent to which hospice and palliative care in nursing homes has changed due to the COVID-19 pandemic, which problem areas have been perceived from multiple perspectives and how the pandemic has affected residents, relatives and employees.

## Materials and methods

The effects of the COVID-19 pandemic on hospice and palliative care in nursing homes were analysed from different perspectives as part of the study on “Hospice and palliative care in nursing homes”.

The study was funded by the Stiftung Wohlfahrtspflege NRW welfare foundation (grant number SW-620-6935) and was initiated in May 2019 with a term of three years (plus six months due to the COVID-19 pandemic) by the project manager Contilia Pflege und Betreuung GmbH. The investigations and analysis were conducted by two independent research institutes without the involvement of the project manager.

As a sample for those analyses, two nursing homes were selected among the facilities of Contilia Pflege und Betreuung GmbH. The criteria for inclusion were general nursing homes, regularity of palliative care, employment of palliative care nurses, location in North Rhine-Westphalia, Germany, and willingness to participate in the study. Nursing homes with a certain specification (e.g., only for residents with dementia) were excluded. To incorporate variation, the selected facilities differed in their location, size, degree of digitalisation, degree of implementation of palliative care and catchment areas of the residents (further details about the differences can be found in [41]).

In the discussion of both the methodology and results, three nursing homes of an extended group of providers as well as an expert advisory board were involved to assess the transferability. The initial aim was to investigate the status quo of palliative care in everyday practice from different perspectives, identify burdens, uncover weaknesses and develop improvement strategies. Those, subsequently, were supposed to be tested and evaluated within everyday palliative care practice and adapted for use in other facilities to meet the overall goal of improving palliative care in nursing homes.

However, as nursing homes were hugely impacted by the effects of the pandemic [20], changes in workflows to implement palliative care improvement strategies could not be undertaken. Simultaneously, significant uncertainty has arisen about the impacts of the COVID-19 pandemic. As the pre-pandemic analyses had already been completed, this study offered unique potential as a baseline to investigate changes and effects of the COVID-19 pandemic. Therefore, the objectives were adapted to offer insights into the effects of the COVID-19 pandemic on employees, residents and relatives as well as their perceived problem areas and fields of action within hospice and palliative care nursing homes.

For this, qualitative data were collected using semi-structured interviews with employees, residents and relatives before [T0] and during the COVID-19 pandemic [T1].

Semi-structured interviews were chosen as a well-established qualitative research method that allows in-depth explorative analyses of the perceived problem areas, fields of action and pandemic changes. This method allows a degree of openness and flexibility for the responses of the interviewees, which was crucial, as not much is known about the perspectives of residents and relatives in particular. At the same time, those interviews allow guidance through certain topics related to the research question. This structure helped to address similar subtopics to the groups while remaining feasible within the framework of the research design. Furthermore, oral interviews were considered most appropriate to incorporate the vulnerable group of residents. Difficulties in completing written surveys were thus prevented.

The interview topics at T0 focused on insights into hospice and palliative care in nursing homes, how hospice and palliative care is carried out and perceived problem areas. The analyses served as a baseline from which pandemic-related changes were investigated. Hence, the focus at T1 was on the perceptions and changes due to the COVID-19 pandemic. Table 1 provides an overview of the topics that were addressed within the interviews.

**Table 1:** Interview topics at T0 and T1.

**Employees**	
T0	Organisational structures of the nursing home in the context of hospice and palliative care Processes of everyday hospice and palliative care practice Cooperations in the context of hospice and palliative care Dealing with palliative residents and relatives and their needs Dealing with the last phase of life, dying and death Workload and burdens in the context of hospice and palliative care
T1	Perceptions of the nursing home dealing with the pandemic and changes of organisational structures in the context of hospice and palliative care Processes of everyday hospice and palliative care practice during the pandemic Providing palliative care under pandemic conditions Changes within cooperations in the context of hospice and palliative care during the pandemic Changes in dealing with palliative residents and relatives and their needs during the pandemic Dealing with the last phase of life, dying and death during the pandemic Workload and burdens in the context of hospice and palliative care and changes during the pandemic
**Residents**	
T0	Physical/psychological symptoms and well-being Individual wishes and expectations on (hospice and palliative) nursing care and their integration by caregivers Self-determination within medical and (hospice and palliative) nursing care Communication, information and processes of (hospice and palliative) nursing care Social relationships and inclusion of relatives within (hospice and palliative) nursing care Dealing with the last phase of life, dying and death
T1	Well-being during the pandemic and changes in physical/psychological symptoms Perceptions, experiences and impacts of the pandemic Perceptions of the nursing home and caregivers in dealing with the pandemic Individual wishes and expectations on (hospice and palliative) nursing care and their integration by caregivers during the pandemic Self-determination within medical and (hospice and palliative) nursing care during the pandemic Sources of information and modes of communication during the pandemic Social relationships and inclusion of relatives within (hospice and palliative) nursing care during the pandemic Dealing with the last phase of life, dying and death during the pandemic
**Relatives**	
T0	Perceived well-being of the residents Communication and information with caregivers, doctors and cooperation partners Processes and inclusion within (hospice and palliative) nursing care Wishes and expectations on (hospice and palliative) care and their integration by caregivers Perceptions on wishes, ideas and self-determination and their integration in (hospice and palliative) nursing care from the resident’s perspective Dealing with conflicts Dealing with the last phase of life, dying and death
T1	Perceptions, experiences and impacts of the pandemic in context of (hospice and palliative) nursing care Communication and contact with residents during the pandemic Communication of information with caregivers, doctors and cooperation partners during the pandemic Changes in wishes and expectations of (hospice and palliative) nursing care and their integration by caregivers during the pandemic Perceptions of the nursing home and caregivers dealing with the pandemic Provided support for residents and relatives by the nursing home during the pandemic Dealing with conflicts during the pandemic Dealing with the last phase of life, dying and death during the pandemic

To obtain access to (palliative) residents and relatives of palliative residents as well as employees, interviewees of each group were recruited by study members in the facilities. Employees, who regularly had contact with palliative residents during their working day and were willing to participate, were included. Residents and relatives were selected by their ability and willingness to participate in the interview and to provide consent. Residents in need of palliative care and relatives with residents who needed palliative care were prioritised. Interview appointments with interviewees who received a verbal and written explanation of the study’s objectives and voluntarily signed an informed consent form were arranged by study members in the facilities.

The acquisition period prior to the COVID-19 pandemic (T0) took place from October 2019 until February 2020.

The second data acquisition (T1) was performed analogously to the first and took place in the period between May 2022 and June 2022. This period was selected because coronavirus restrictions had been loosened the month before in the state of NRW based on a new coronavirus protection regulation and changes to the German Infection Protection Act [42, 43]. Thus, interviews could be conducted in person. Where possible, the same participants were interviewed. However, due to staff fluctuation and the deaths of residents, this was not possible in all cases. Furthermore, the pandemic led to difficulties in recruiting an according number of interviewees in the groups of residents and relatives. The following table provides an overview of the sample number and characteristics at T0 and T1 (Table 2).

**Table 2:** Interviewee characteristics.

Perspective	Criteria	T0	T1
**Employees**	N	14	14 (8*)
	type	caregivers: n = 10, manager: n = 4	caregivers: n = 10 (6*), manager: n = 4 (2*)
	sex	female: n = 11, male: n = 3	female: n = 10, male: n = 4
**Residents**	N	14	10 (4*)
	sex	female: n = 10, male: n = 4	female: n = 9, male: n = 1
	mean age	85 years	86 years
	distribution of care level	level 2: n = 4	level 2: n = 1
		level 3: n = 5	level 3: n = 4
		level 4: n = 5	level 4: n = 3
			level 5: n = 2
	mean length of stay in the nursing home	3 years	3,5 years
	in need of palliative care	n = 6	n = 4
**Relatives**	N	14	10 (1*)
	sex	female: n = 7, male: n = 7	female: n = 8, male: n = 2
	mean age	66 years	70 years
	mean age of the related resident	94 years	90 years
	mean length of related residents stay in the nursing home	3,7 years	3,3 years

*Number of same interview partners as at T0

**Fig 1:**
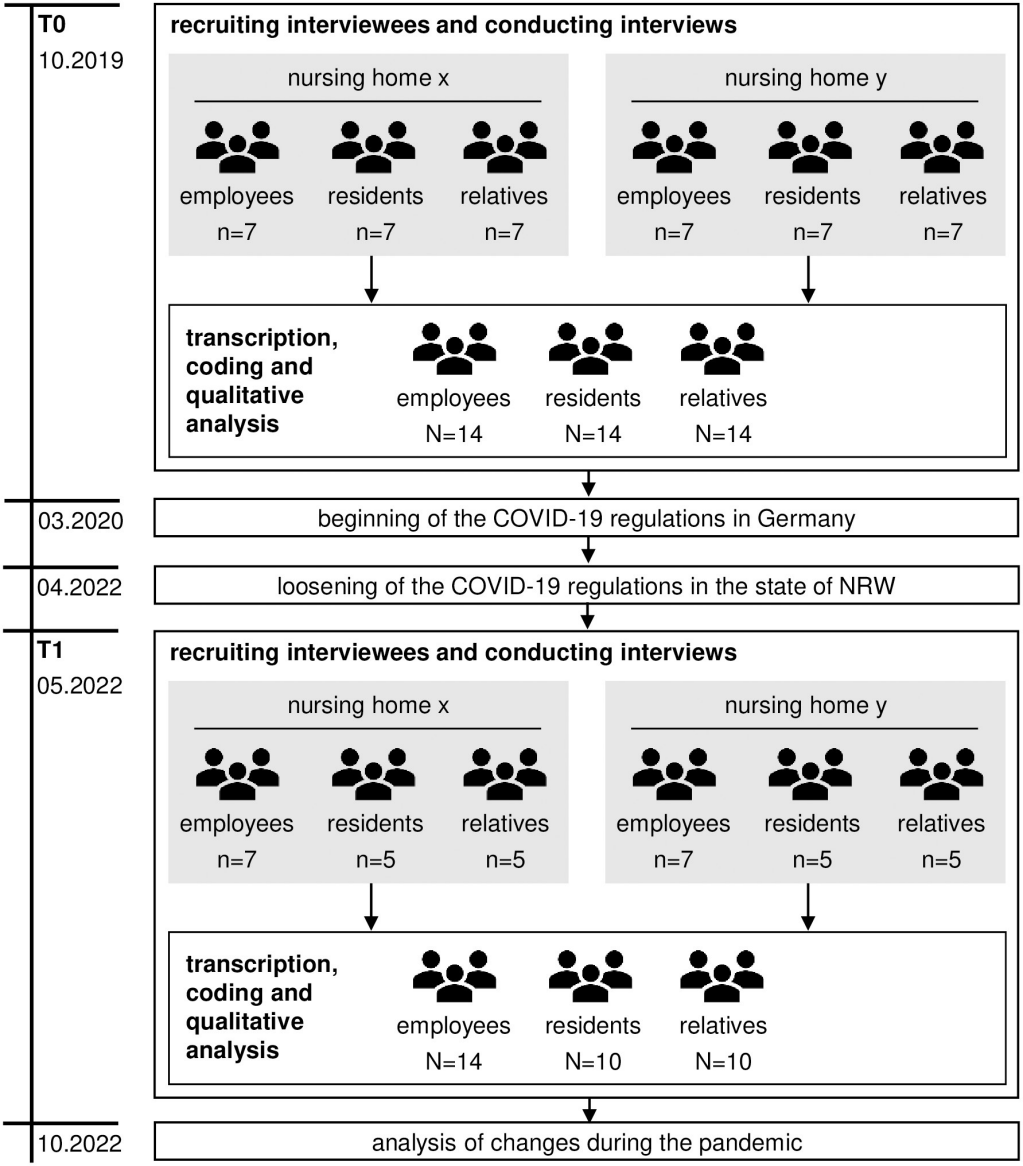
Flowchart of the study design.

The interviews were recorded and transcribed with the consent of the interviewees. The evaluation took place pseudonymised using MAXQDA software following the content analysis method according to Mayring [44]. According to the addressed interview topics (Table 1), the code system was first deductively defined and passages relevant for the analysis were extracted from the transcripts and coded [44]. The category system then underwent a final inductive adaptation based on the categorised text segments. This was followed by a final material run-through with a double check of two evaluators [44]. After coding, the extracted material for each category was summarised with regard to subjectively perceived problem areas and fields of action concerning hospice and palliative care [44]. Finally, the results were analysed in a pre-post comparison of the effects of the COVID-19 pandemic on hospice and palliative care. An overview of the study is provided Fig 1.

The study was performed with written approval by the Ethical Committee of University Siegen, Germany (ER_24/2019) who was not involved in the study. All participants participated by choice without incentives for participation, with the exception that the survey of the employees was considered paid working time. All participants had the option of refusing to participate or to withdraw their participation at any time. Confidentiality was assured.

As interviews were conducted in German, quotes in the results chapter are translated. The original quotes can be found under S1 File.

## Results

### Employees

From an employee perspective, the following subjectively identified problem areas and fields of action as well as the effects of the COVID-19 pandemic on hospice and palliative care were subsumed in survey periods T0 and T1. Findings from the T0 survey period, as a baseline, are summarised in Table 3 and can be looked up in more detail in [45].

**Table 3:** Employee perspectives at T0.

Employee perspectives at T0	
**Identification of the need for palliative care** Predominantly based on subjective values of employees in their daily routine No use of assessments Reliable identification questionable Problematic for inexperienced employees who expressed uncertainties in this respect	**Exemplary quotes** *“For me it is not quite clear when we actually become active now*. *Of course*, *if there is a change in health status and we see ‘oh*, *someone is about to leave’ but I find that it would have to be better clarified*. *That would give me security*. *[*…*] That is actually not organised yet*, *I think*, *yes*? *That makes me a bit insecure*, *that I have to look for it myself*, *so to speak*.*” (MA_2)*^*1*^ *“But sometimes we are simply wrong*. *[*…*] Sometimes it is more a bit of trial and error*.*” (LF_3)*^*2*^ *“Sometimes I think that is maybe also because I am still very young*. *Maybe that comes with experience*, *I hope so at least*. *But like I said*, *I hope*, *maybe I’ll learn that when I do the palliative care*, *the specialised training*. *Maybe I’ll learn that*. *I hope*.*” (MF_3)*^*3*^
**Collaboration with cooperation partners** Desire for greater availability of medical staff and stronger on-site presence Different perception of cooperation partners and nursing home staff with regard to the urgency of situations in the palliative phase Unstructured involvement of outpatient hospice services based on the perception of employees	**Exemplary quotes** *“Well*, *what I really find fault with is the way we are set up with doctors*, *the medical support in the palliative area*. *I would like to see that improved*. *Just for cases like case conferences*, *ethical case discussions and something like that*, *consultations*. *I’ve hardly noticed that you really have reliable support from the doctors and sometimes I think that’s urgently necessary*.*” (MF_2)*^*4*^ *“With the doctors*, *sometimes they don’t see the urgency like that*, *like*, *‘Yes*, *I’ll come tomorrow*, *the day after tomorrow*.*’ ‘No*, *now*. *Today*. *Tomorrow he might already be dead*.*’ Or when they say*, *‘Yes*, *he’s in the palliative phase*,*’ but they don’t prescribe Tavor or morphine*. *‘In case of need*, *you have something there*.*’ ‘No*, *that’s theft*. *We are not allowed to take something from another resident*.*’ (LA_4)*^*5*^ *“[*…*] but the people who get into the dying phase*, *they want familiar faces*. *So when someone from the hospice comes*, *they are strangers*. *You don’t even know if there is sympathy; he doesn’t know me at all*. *So the offer is actually little taken up [*…*]*.*” (MA_4)*^*6*^
**Uncertainties in the daily work routine** Uncertainties in palliative care processes due to a lack of structural and organisational specifications Uncertainties in the coordination of time-critical palliative processes and activities Uncertainties in dealing with relatives of palliative residents Uncertainties with respect to when to focus on care and human attention rather than on routine care processes in the palliative phase	**Exemplary quotes** *“I know that certain processes take place*. *I doubt that they are really always regulated in such a way that they take place in an orderly manner*, *and I know for myself that if these regulations should exist*, *I have not yet been able to internalise them or they have not yet been brought to me*. *I think many of us feel that way*. *[*…*] My impression is*, *also because of my own insecurity*, *somehow I have my doubts whether I am always so aware of how it should perhaps be done*.*” (MA_2)*^*7*^ *“[*…*] you have never gone through this before*. *How do you behave*? *What is the procedure here*? *I remember when I started here*, *someone was in the phase and then died during the handover and I didn’t know what to do*.*” (MF_5)*^*8*^ *“Yes*, *then the full care or shower just falls away*. *[*…*] Because the person says*, *‘I am not able to do more and I don’t want more*. *But I am clean*.*’ These are things where you really have to look and say*, *‘Yes*, *that’s okay*.*’ But the staff really have to get that and it has to be implemented*. *But you will notice that they have difficulties with that*. *One more*, *the other less*.*” (LF_2)*^*9*^
**Stress in the context of palliative care** Sparse time resources for hospice and palliative care as the greatest stress factor Stress due to guilty conscience Stress due to relocations of dying people to the nursing home on short notice Uncertainties in the transition from care processes in the palliative phase as a significant stress factor Stress due to the absence of resources, such as medication Stress due to non-specific arrangements and/or unclear medication Stress due to potential conflict with relatives Palliative care was described as exhausting work	**Exemplary quotes** *“So*, *I can say for myself now*, *I don’t always go home with a good feeling and think that all my residents are now well looked after overnight*, *no*, *I don’t but it would*, *it would be desirable*.*” (LA_3)*^*10*^ *“I sometimes have difficulties when people are*, *I say this very badly*, *discharged from the hospital*. *When they have two or three days left where I think to myself*,, *‘Where is the humanity*? *Where is the human being*?*’ [*…*] Often they come without medication and I get crazy*. *Or it was not given along with or they have forgotten it*. *[*…*] In those situations we are really desperate*.*” (LA_2)*^*11*^ *“In itself*, *the burden is being confronted with suffering and death here*. *[*…*] It’s not easy*, *and it’s also not easy to work in an environment where you actually have to deal very*, *very strongly and continuously with suffering*, *dissatisfaction*, *fears and all the dark thoughts*. *To always want to counter this with something positive is sometimes exhausting*. *And also the awareness that you can’t always do it*. *It is a very energy-sapping activity*.*” (MF_2)*^*12*^

#### Problem areas and fields of action from the perspective of employees at T1

The findings from the interviews in the T1 survey period illustrate that the pandemic-related adjustments and hygiene precautions presented a number of challenges for employees and managers within the scope of hospice and palliative care.

In particular, palliative care with personal protective equipment was considered stressful by employees, as the emotional component with direct skin contact was missing. Furthermore, in some cases, employees were no longer recognised by residents, and residents responded to the protective clothing with anxiety and the mask-restricted communication.

*“[…] you couldn’t just sit on a bed and hold a hand or even just stroke a face […]*. *Doing something in gloves and a gown is completely different*, *and the person has no skin contact*.”

*(ME_6)* ^13^

*“For instance*, *they don’t even know whether a man or woman has entered […]*. *You have a face shield on*, *glasses*, *a hair net*, *gown and a mask*. *And communication is almost impossible*. *If you are working with someone with poor hearing*, *it’s even worse*. *People with poor vision and those with dementia no longer understand what’s happening*.”

*(ME_7)* ^14^

The effects of visitor restrictions during the pandemic were described as a further challenge. Although exceptions were put in place in the final phase for palliative residents and their relatives to say goodbye, end-of-life care was significantly restricted during this period.

Employees also described difficulties in reaching doctors and less on-site presence of medical cooperation partners during the pandemic.

*“Until you had the doctors on the phone*, *we really had a lot of work to do*, *writing faxes and saying ‘We need you*. *Call back*.*’ It was really bad*. *[*…*] I found it more extreme because before you could actually get them on the phone relatively often*. *But since the beginning of the pandemic*, *it’s really a no-go*. *I think it’s really bad*.”

*(MM_6)* ^15^

At the same time, group care offers were replaced with individual care services with the same time and staff resources, which reduced the care time for the individual residents, hampered palliative care and was not satisfactory to employees.

*“Naturally*, *this led to a certain drop in quality for palliative residents*. *After all*, *you want to look after everyone and keep them happy*.”

*(MM_1)* ^16^

Additional hospice care and pastoral care offers were also suspended temporarily. The restricted visiting opportunities for relatives, the reduced care and the suspension of hospice care by third parties led to employees identifying depressive changes in residents. Employees therefore tried, wherever possible, to compensate for the lack of social contact of palliative residents, especially in their final phase. Although it was reported that this took additional time, employees and managers also highlighted that this situation brought them closer to palliative residents and intensified the relationship.

*“In a sense*, *they acted as family surrogates*, *which they did not actually want to be*, *should not be and cannot be*. *[…] But in these exceptional circumstances*, *we naturally provided a bit more leeway*. *Even we as managers tolerated greater proximity*, *which we permitted between our employees and the residents*.”

*(ME_1)* ^17^

Although the opportunity for video calls was provided in the project nursing homes, employees reported that the calls had to be supervised for the technical handling and that the benefits for residents were partially low. Employees also described that they sometimes found video contact stressful for residents.

*“And especially this*, *‘Yes*, *it’s great that you are sending photos*. *But the resident can’t do anything with them*.*’ Or*, *you send a video and the resident has the feeling that you are live and chats with you*. *They don’t really realise what it is*. *So then you sometimes had the feeling that this is having more of a negative rather than a positive impact*. *We also had three residents who always immediately started crying as soon as they saw their daughter or their son on the tablet or had them on the phone*. *You had to say*, *‘It’s just not possible at the moment*. *We need to stop you*.’

*(ME_5)* ^18^

However, with the exception of requests for visits during the contact restrictions, the majority of those surveyed reported that the requests and needs of palliative residents were able to be met.

*“Oh*, *I think they meet them [resident requests]*. *And*, *if not*, *they try to do everything possible to meet the requests and needs*. *Absolutely*. *I think it works well*. *Yes*.”

*(ME_2)* ^19^

Due to visitor restrictions, dealing with relatives was described as problematic. First, a lack of on-site presence made contact and information exchange difficult, and, second, relatives responded to pandemic-related restrictions with different levels of understanding.

*“Some were very understanding*, *and some had absolutely no understanding and screamed and cried at the entrance to the nursing home*, *blaming us for not being able to see their mother*. *Threatened us with the police and all sorts of things*.”

*(ME_1)* ^20^

However, some also reported an intensification of the relationship with relatives during the pandemic.

Employees tended to be of the opinion that more time was required due to a greater need for communication by relatives.

*“After all*, *they also had a need for communication; relatives would call*, *and they needed to be informed and reassured and everything as well*. *[…] And this certainly took additional time and effort*.”

*(MM_1)* ^21^

However, one person recounted that the time taken to deal with relatives fell due to visitor restrictions.

Employees and managers described that the pandemic situation caused uncertainty due to adjustments within the nursing homes at short notice due to the pandemic, media reporting and the in-house handling of palliative residents so as not to expose them to any further risk of infection.

*“At the start there was a huge amount of uncertainty […]*. *You have no idea about this damn disease*, *this virus*. *You have no idea how it will behave*. *It seemed like*… *when you drove to work*, *lockdown*. *The streets are empty*, *and then you come trundling along*. *And then (shhhh) the door opens and (shhhh) and you go and get started*.”

*(MM_1)* ^22^

Uncertainties resulted in a balancing act between wanting to provide the aforementioned compensation for the lack of social contact through a stronger staff presence and avoiding the rooms of palliative residents, which was perceived ambivalently by the employees.

*“[…] There was a fear that you might somehow pose a risk to palliative residents or infect them*. *[…] I think that you may have stayed for a shorter period than you perhaps sometimes needed to*.”

*(ME_3)* ^23^

*“And returning to the room*, *there would be no one in the room for the next three hours*. *So I had to just look into the room 4*, *5*, *6 times because no one is present*. *That increased*, *yes*.”

*(ME_7)* ^24^

Uncertainties in identifying the need for palliative care, the assessment of palliative symptoms and with regard to the process and communication channels were less of a concern for employees than for managers.

*“Quite uncertain*. *[…] Yes*, *I think that they were afraid to assess someone as palliative or that they would not be able to assess them correctly*. *[…] I have come across situations where it’s*, *‘What*? *What is your assessment*?*’ I ask*, *but they are just completely uncertain*.”

*(ME_2)* ^25^

However, in some cases, employees expressed a desire for more information on structures and processes in hospice and palliative care.

*“[…] To clarify what’s new in palliative care […] or what is currently going on in the nursing home*. *It’s true that we were always involved in the meetings*, *but I am not sure whether everything was always communicated or whether there might have been a thought in retrospect*, *‘And oh*, *I should have said that*.*’ I would find a kind of palliative afternoon that also provides information about the residents good*.”

*(ME_3)* ^26^

As a positive outcome, managers reported that the identification of palliative care situations improved, even though the assessment continued to be based on the empirical values of employees without the use of validated screening instruments.

*“It is now much clearer as to who is in a palliative care situation and how the situation currently stands*.”

*(ME_1)* ^27^

In the interviews, employees and managers repeatedly described greater stress due to multifactorial influences brought about by the pandemic. Time and staff resources were identified as the greatest stress factors, which escalated during the pandemic. Reasons for this were staff absences due to isolation and illness together with a rise in time and effort associated with the pandemic as a result of testing, protective measures, the takeover of more personal services and the compensation of social contact.

*“Care and support is always associated with time pressure*. *[…] But this obviously increased even further*. *[…] Even just due to the number of times that colleagues had to disinfect and wash their hands*, *if you extrapolate that you already get value that is then missing in effective care*.”

*(ME_1)* ^28^

*“So*, *we provided foot care*, *hairdressing*, *pastoral care and were family that could just give them a quick hug and listen*. *[…] And*, *well it all certainly needed to be managed*.”

*(MM_4)* ^29^

Just under half of respondents indicated that they were not able to meet their own standards of hospice and palliative care. In some cases, respondents added that they could not manage the high workload. Due to the increase in the scope of work, the majority of respondents described more time pressure as well as the necessity of daily prioritisation of tasks. This impaired other activities, which also impacted the time spent with residents.

*“When you work under these conditions*, *you are forced to prioritise virtually every day*. *What is an absolute priority today*? *What do I need to get done*? *What is a clear necessity and what can I postpone*?”

*(ME_1)* ^30^

*“[…] I do not have the opportunity to sit beside residents*, *switch off and be entirely present just for the resident*. *Personally*, *I was not able to do it*. *In my heart*, *maybe*, *but not in my head*.”

*(ME_6)* ^31^

An additional stress, particularly at the start of the pandemic, was the fear of infection with the SARS-CoV-2 virus at work and infecting a person’s own social group or palliative residents. However, over time, half of employees reported that the risk of infection at work no longer had an impact on themselves personally or their private life.

However, respondents also reported their ongoing sadness and sympathy for residents having to endure their end of life under pandemic conditions. Furthermore, they indicated a feeling of helplessness at the speed of increase in the symptoms and deterioration of the general condition of residents infected with COVID-19.

*“And I have no idea how we will be able to do this kitted-up after the pandemic; after all*, *at some point there will be a post-pandemic period*, *because of the circumstances under which people died in some cases*. *The rapid speed was unprecedented*. *Anyone who has not had to stand by a bed in these moments would not believe it*. *[…] And there were so many tears*. *There was despair because you wanted to help but simply could not because […] the necessary technical-medical requirements were not in place*.”

*(ME_1)* ^32^

### Residents

The survey of residents revealed the following topics and fields of action at baseline (T0), listed in Table 4.

**Table 4:** Resident perspectives at T0.

Resident perspectives at T0	
**Well-being and care** Most residents reported feeling stressed and/or sad Residents indicated that they were largely satisfied and did not have any specific requests. There often was a lack of comprehension of the question, as the possibility of expressing a request was viewed as absurd If there were requests, they tended not to be expressed (“It won’t be met, so I don’t ask, that’s what I have learned in my life”) If requests were expressed, they tended not to be met The topic of eating played an important role in the meetings: many were not satisfied with it and/or felt shame due to unseemly behaviour as a result of problems with teeth, shaking hands or impaired eyesight	**Exemplary quotes** *“Sad I am constantly*.*” (BA_3)*^*33*^ *“They always ask what d/you want*. *I say nothing*. *I am happy having my water*, *my air to breathe*, *my newspaper and my visits*.*” (BA_4)*^*34*^ *“One resident always gets cheese instead of jam in the morning*.*” (BF_5)*^*35*^ *“Yes*, *yes*, *she’s got problems with her teeth*. *During eating she takes them out*, *putting them on the table*, *and the other one*, *she pulls out the rests after eating; that’s why I prefer eating in my room*.*” (BF_3)*^*36*^
**Participation** Approximately one third had good contact with other residents Conversation partners in the nursing home were predominantly carers, who had very little time In general, residents tended to report that they had few (potential) conversation partners With respect to nursing home activity offers, there either was no demand (no matching offers/no interest) or a very strong demand	**Exemplary quotes** *“I have lots of contact with other residents*.*” (BA_7)*^*37*^ *“No*, *there is nobody I can talk to*. *(…) Well*, *yes*, *‘why or why not…’ is not being asked*, *they don’t have any time there*.*” (BF_2)*^*38*^ *“No*, *there are offers*, *but that is mostly for those who are not so fit any more and cannot think clearly I don’t feel like sitting down and playing a game [called “Mensch ärgere Dich nicht”] or so…” (BA_5)*^*39*^ *“Yes*, *sure there are enough offers*. *I don’t participate in all of them but in a lot*, *right*?*” (BF_5)*^*40*^
**Dealing with dying and death** Residents deal with the last phase of life as well as dying and death differently (repression, calmly awaiting, intensive preoccupation (incl. in dreams), longing for death) Development of the idea of a “good death” from experiences with the deaths of other people (relatives and friends) Requests and expectations (most common: wanting to die alone or in company, it should be quick, composedly awaiting death) Residents essentially assume that they cannot have any influence on their last phase of life Only few specific organisational requests are defined for the last phase of life There are virtually no discussions on specific requests and expectations concerning the dying phase with relatives as well as employees In discussions about the end of life and death in general, communication with the family takes priority The possibility of hospice care is a virtual unknown, unless this has been independently experienced by relatives or friends Nursing home rituals after residents have died are known in some cases (black ribbon and book of condolence) Possibilities of saying goodbye to deceased residents are not well known Formal matters (e.g., funeral modalities) are regulated	**Exemplary quotes** *“I just want to get up*, *turn over and be gone like my husband*.*” (BA_7)*^*41*^ *“I look at it easily because we can’t change it*.*” (BF_5)*^*42*^ *“No*, *that our children will manage; we sorted that out with our children*.*” (BA_1)*^*43*^ *“…want to die all by myself*. *I don’t need anybody with me… yes*, *Jesus*, *he guards me*.*” (BF_4)*^*44*^ *“I haven’t heard anything of that [hospice care]*.*” (BF_7)*^*45*^ *“Yes*, *well the relatives*, *they come*. *They are allowed again*, *but us*, *we are not allowed into the rooms*.*” (BA_5)*^*46*^

#### Problem areas and fields of action from the perspective of residents at T1

For the majority of those surveyed, the pandemic was not a defining, negative experience. Most endured it more or less composedly. One person indicated that they had no negative recollections of the pandemic, while another could not remember it at all.

*“No*, *that doesn’t mean anything to me*. *It passed by*, *no absolutely nothing*, *nothing*, *there were no restrictions*. *[…] I was vaccinated at some point but I am not sure what it was*.”

*(BE_1)* ^47^

Despite being vaccinated, three people caught COVID-19 and spent up to three weeks in quarantine. This, too, was endured more or less composedly and without great fear.

*“It was bad but I survived (laughs)*, *but no fear*, *I had nothing in that respect (*?*)*”

*(BE_2)* ^48^

The approach to the pandemic by the nursing homes received positive assessment by the residents. In general, residents felt that the way the nursing homes approached the pandemic, was appropropriate. One person even felt safer in the nursing home compared to “outside”.

*“After all*, *we are very protected here aren’t we*? *For instance*, *when the numbers increased outside*, *we were kind of like on an island*.”

*(BM_4)* ^49^

The acceptance of the measures was high across the board.

*“Well*, *that’s how it has to be*, *otherwise it will never end*.”

*(BE_2)* ^50^

Even if they were found to be stressful in some cases. For example, with regard to respirator masks, it was reported that it was difficult to breathe and that strangers/people were difficult to recognise. One person stated the following concerning the vaccinations:

*“After all*, *you can’t just say*: *‘No*, *I am not doing that’*, *when you live in society you have to integrate don’t you*?”

*(BM_4)* ^51^

Even severely restricted social contact was met with understanding.

*“[…] and at the time we were able to walk onto the balcony and have a chat*, *I mean you had to show understanding*, *they did not want to have any difficulties*, *the relatives*.”

*(BE_3)* ^52^

None of the respondents stated that their self-determination was not ensured or that they did not agree to the measures, such as vaccinations. There were different perceptions as to the extent to which social contact had to be restricted. The majority of the surveyed residents expressed that personal contact was no longer possible. One person described this as stressful.

*“[…] we all had to be by ourselves*, *no one was allowed to join me*, *yes it was terrible*.”

*(BE_2)* ^53^

However, two people also indicated that they had regular visitors, but they were not allowed to hug. While this was accepted, it was found unpleasant. In general, (greater) use was made of the telephone to maintain contact with relatives, but one person reported that they explicitly did not want this.

When asked what they would have liked to have been different during the COVID-19 period, a number of residents referred to in-person visits by relatives. Two people provided very unassuming responses, with one indicating that there were sacrifices that had to be accepted.

The ability to be comfortable spending time alone was found to be helpful during the COVID-19 period. This was supported by certain activities, such as crafting, reading or spending time on the computer.

*“Yes*, *I played a lot of music and killed some time with the computer or television*. *Yes*, *I always tried to keep busy; you can always find something (laughs) that you can do*.”

*(BE_3)* ^54^

Requests concerning care and support tended not to be expressed, and if they were, they were of a more general nature.

*“That I am not getting worse*, *that I may be getting better or that things are good as they are now*, *nothing more*.”

*(BM_3)* ^55^

Whenever minor requests were expressed, most remained unmet.

*“I get on well with everybody*. *If there is something I do not like*, *I tell them and I understand if I have to lie here for an hour before someone arrives*. *I have previously requested a cocoa*, *but instead there was broth; my wife will say something when she comes*.”

*(BM_1)* ^56^

In addition, residents lacked points of contact among the employees.

*“An entirely different situation should have been created*, *so that there is at least someone that you can talk to and who is familiar with the patient*. *This was all pushed into the background*, *the main thing was that the nursing home is running and money is flowing; the rest is not important*.”

*(BM_3)* ^57^

The surveyed residents overwhelmingly reported that they did not think about the last phase of life or deliberately pushed the thought away.

*“No*, *no*, *not yet*. *I push them away*. *Yes*, *I don’t think about them or think*, *‘OK*, *there will be a day when one of your relatives dies and it could be you*, *but God does not want you yet*.*’ That’s how I think*, *and then it’s all passed*. *It’s unpleasant for me; I don’t want to think about it*. *I think*, *when your time comes*, *then it comes*.”

*(BE_1)* ^58^

In terms of the organisation of the last phase of life, there was a great reliance on relatives.

*“First*, *the time has to come*, *and then my wife will know what to do*.”

*(BM_1)* ^59^

*“No*, *honestly*, *I don’t think about it; I rely on my children*.”

*(BM_2)* ^60^

Most respondents assumed that they would not be alone at the end of life. Above all, having trusted family members by their side was most important.

*“When I’m feeling poorly*, *the children are here; they come*. *My husband is with me; he won’t leave me alone and the children will come*, *however they can*.”

*(BE_1)* ^61^

Two people stated that they would prefer to die alone. One described that their feelings in this respect had changed and that they would now find it unpleasant if everyone were standing around the bed at the end of their life. The other person stated that they did not want to burden the children and so had to “do it” alone.

Even if requests (e.g., not wanting to be alone) existed, they tended not to be expressed to employees and relatives.

*“No*, *I have not said anything yet*. *I think it is a decision that the children should also be part of*, *shouldn’t they*? *Whether that will be the case*, *I don’t know*, *I don’t know*. *I think that it is something you cannot really plan; it happens when it happens*.”

*(BM_4)* ^62^

When asked what they would have liked done differently during the pandemic, the residents once again had quite an unassuming and pragmatic attitude.

*“It is no longer how it used to be; you have to make some sacrifices*. *We used to have a small choir*, *but that has stopped*. *And many of those involved have died*. *That is certainly a difference here*.”

*(BM_3)* ^63^

There was an overwhelming desire for more family visits.

*“Yes*, *just that the children could come in*. *But that couldn’t happen*, *could it*? *It was not possible*.”

*(BE_2)* ^64^

*“The only thing that I missed was personal family visits*. *Otherwise*, *I wouldn’t know anything else; things kept rolling along in here*.”

*(BM_5)* ^65^

### Relatives

Baseline problem areas and fields of action perceived by the relatives at T0 are summarised in Table 5.

**Table 5:** Relatives’ perspectives at T0.

Relative perspectives at T0	
**Organisation and communication** Communication with carers predominantly took place during visits to the nursing home and only at the initiative of relatives as impromptu conversations There were no regular communication structures in a relaxed atmosphere, only the ad hoc clarification of questions/problems due to a lack of employee time as well as lack of continuity of staff No contact with other people involved in the care. Relatives had no information of the care and support provided by other professionals; the responsibilities were not clear Relatives had no “point of contact” for their concerns with respect to hospice and palliative care The needs of relatives were not taken into account by the nursing home	**Exemplary quotes** *“The caregivers do not actively come up to me as a relative and say*, *‘Your father is now taking this medications*,*’ or ‘Today the doctor was there and this has been changed*,*’ or ‘Your father has some new complaints*.*’ It‘s not like that*.*” (ZF_2)*^*66*^ *“In some cases*, *a different nurse is there every day*. *How are you supposed to have an exchange there*?*” (ZA_1)*^*67*^ *“I know that a therapist comes to my husband but I don’t know what she does or whether he has participated or not*.*” (ZA_5)*^*68*^ *“The neurologist*, *who comes to the home*, *had not even allowed me to participate in a conversation*. *When they changed doctors*, *I wanted to tell them something about my mother and get to know the neurologist*, *but that’s not possible*.*” (ZF_4)*^*69*^
**Involvement in care and support** Relatives were most likely to contribute when it came to the interior design Their own ideas were not communicated, although the desire existed because: structures were found to be too rigid to introduce certain matters the physical condition/health of the resident was poor and relatives had no idea how they could get involved there was a fear of financial consequences There was no guidance, advice, or support by employees Opportunities for active contributions to the last phase of life were not known The involvement of relatives in the last phase of life was not addressed in advance (neither by residents nor employees)	**Exemplary quotes** *“So the room that my mother has now*, *I hung up a few pictures myself*.*” (ZA_2)*^*70*^ *“I know that for some people*, *I am exhausting and annoying*, *and of course they find it exhausting that I get involved so often*. *Of course that doesn’t suit them*.*” (ZA_1)*^*71*^ *“Yes*, *but I think they have a structure*, *it’s predefined*, *and I didn’t have the feeling that I could somehow contribute something*.*” (ZA_6)*^*72*^ *“I was told that I could accompany her when the time came*, *but so far it hasn’t come yet*. *I don’t know how that will work out and what you can do then*. *I have no idea*.*” (ZA_2)*^*73*^
**Dealing with the last phase of life** Importance of participation in care and support increased in the last phase of life. Involvement was presumed by relatives Great uncertainty existed amongst relatives in dealing with dying and death There was virtually no communication on the last phase of life with residents and employees. Reasons included: No awareness/lack of knowledge that something could be discussed Fear of addressing the topic No need (yet), as the resident was faring well Communication desired but not (no longer) possible (due to condition of the resident or lack of employee time) The possibility of integrating outpatient hospice services was largely unknown and unused Respite services offered by the nursing home were unknown, and employees did not convey external offers No detailed knowledge of the process surrounding deceased residents	**Exemplary quotes** *“I think I will be involved*. *They have my cell phone number and will certainly call me*.*” (ZA_4)*^*74*^ *“That’s not really what we talked about*, *how she thought it was going to be*.*”(ZA_2)*^*75*^ *“We didn’t talk about it in detail*. *Yes sure*, *burial*, *cremation*, *things like that*, *but we didn’t talk about how it should end*.*” (ZF_5)*^*76*^ *“There are definitely rituals here*, *but I can’t give you that*, *um*, *um*, *in detail*.*” (ZF_2)*^*77*^

#### Problem areas and fields of action from the perspective of relatives at T1

During the visitor restrictions during the pandemic, contact between palliative residents and their relatives predominantly occured over the phone or via video calls and, later on, in a park or behind acrylic glass. In some cases, no contact was considered the best solution to avoid confusing residents.

Contact with employees took place over the phone. Overwhelmingly, it was left to relatives to take the initiative, all of whom stated that they could contact employees at any time.

*“[…] I was always able to call*. *I was often hesitant to call*, *but they said*, *‘Don’t be silly; you can call every day*.’

*(ZM_2)* ^78^

Some employees also independently made contact with relatives to inform them of the current situation. However, in the interviews, it was repeatedly highlighted that the initiative was heavily dependent on the specific employee and that no coordinated reporting or information culture existed at the nursing homes.

*“Normally*, *they got in touch*, *but he was also taken to the hospital without calling me first*. *He was taken away*, *and we had no idea where*. *We did not find out where he was*. *I said*, *‘Is he staying there*?*’ ‘We are not sure either*.*’ At some point*, *it was really late at night*, *ten or eleven*, *the night nurse called to say*, *‘He is back now*.*’ This all came out when I suddenly received a report*, *but that was weeks later*.”

*(ZM_3)* ^79^

In some cases, employees considered the needs of relatives during the pandemic. For instance, they were advised to look after themselves more and give themselves a break.

*“At the start*, *yes*, *‘You have to look after yourself*, *you can rely on us*.*’ But this depended heavily on the individual*.”

*(ZE_4)* ^80^

*“Yes*, *they often scolded me and told me that I should take a break or think about myself occasionally and that I did not always need to be at the ready*. *Yes*, *that’s true*.”

*(ZM_2)* ^81^

One relative was referred to the outpatient hospice service due to their high level of stress.

According to the relatives, the experience of palliative residents during the pandemic was overwhelmingly very stressful. In particular, loneliness due to the initial visitor restrictions was stated as a problem for residents.

*“Loneliness was the worst for my husband*. *He cried a lot*.”

*(ZM_1)* ^82^

*“[…] But it was bad*, *often terrible […]*. *We called each other every morning*, *and then it would be*, *‘Just come by*.*’ I would say*, *‘It’s not possible; I am not allowed in the home*.’

*(ZE_2)* ^83^

Those surveyed reported that the lack of care and activity offers in the nursing home during the contact restrictions exacerbated residents’ feelings of loneliness.

The majority of relatives found the time during the visitor restrictions in the nursing homes to be stressful. However, the recollections of interviewees primarily focused on individual, very difficult moments.

*“One time was really bad*: *I wanted to visit in the afternoon*, *but the doors were locked*. *I dropped something off*, *and she was standing behind a glass door*, *and I was on the outside; it was a terrible moment for me and above all also for her*.”

*(ZE_2)* ^84^

However, some of the relatives who visited the nursing home daily prior to the pandemic and never skipped a day due to a guilty conscience reported that, on the one hand, they found the contact restrictions terrible, as they sympathised with the residents. On the other hand, it also provided stress relief, as they were not able to enter the nursing home during that period, making daily, in some cases, energy-sapping visits impossible without having to blame themselves.

The palliative residents to whom the pandemic and the associated protective measures could be clearly explained accepted these more readily, which also made the situation easier for relatives. Other relatives found the enforcement of the measures to be an ongoing battle and an additional stress.

*“Yes*, *I would say that she did not understand*. *She did not understand the entire pandemic*. *Always*, *‘Why are you coming in wearing that rubbish on your face*?*’ Well*, *she didn’t understand*. *You could explain it to her and then she would say*, *‘Well*, *can you take that thing off*?’

*(ZE_1)* ^85^

However, all relatives reported that contact restrictions were necessary to protect residents as well as visitors. They also agreed that the protective effects outweighed the undesirable effects. Overall, the relatives were very satisfied with the management of the pandemic by the nursing homes, as the respondents believed that the situation was taken seriously, the measures were applied consistently and major outbreaks were able to be prevented.

In terms of fulfilling the requests of palliative residents, interviewees felt that the nursing homes were focused on larger, one-off actions, with a view towards “What would the person like to experience again?” This initiative was considered to be very valuable by relatives. However, employees did not consistently and reliably implement smaller day-to-day requests. The fact that this was highly dependent on the individual employee was highlighted once again. For instance, one relative reported that it was important for the independence of the resident that he had his own telephone in his room. However, this resulted in the resident increasingly calling his family, even at night. As he could no longer leave his bed independently, the relative asked the employee to place the handset in a drawer away from the bed at night and replace it in the morning so that he could make calls as needed during the day. However, this was not acted on by all employees. The proposed solution by the staff was:

*“Then*, *you take the handset off him*.”

*(ZM_3)* ^86^

Even in conflict situations, the suggestions of surveyed relatives tended not to lead to changes. In the survey, there were only two groups: relatives who had never had a conflict in the nursing home and relatives who addressed problems but had no effect.

*“Responsibility for conflicts is passed on*. *After all*, *admitting faults is very difficult*, *but we also rely on them so you have to be very careful*.”

*(ZM_1)* ^87^

*“I have gone to see the nursing home management several times and also called*, *but nothing much has changed*.”

*(ZE_2)* ^88^

However, particularly in the context of the pandemic, there was also a stronger fear that addressing conflicts would negatively impact the care relationship in a period where people were even more reliant on employees given the lack of opportunity to visit residents.

At the same time, relatives provided an insight that certain things simply could not be expected, as they could not be provided by the employees.

*“Yes*, *I would like more conversation for the elderly*, *who are just sitting in their room because there is nothing to do; I wish it with all my heart*, *but it’s just not possible because there are not enough staff*.”

*(ZE_2)* ^89^

There was no increase in conversations on organising the last phase of life during the pandemic. The reasons provided were that relatives had not thought about having this kind of discussion or that there was not yet any need, as the palliative resident was still faring well given the circumstances. Another reason was the fear of causing an argument:

*“I don’t like to talk about it with her because she starts to cry*. *When she is having a good day*, *you could talk to her about it*, *but I am scared of it myself*. *It’s so final (cries)*.”

*(ZE_4)* ^90^

Ultimately, some relatives no longer had the opportunity for this discussion due to the health condition of the residents. During the contact restrictions, other relatives did not want to spend the short time on the phone discussing these kinds of difficult topics. In particular, they did not consider a telephone call to be an appropriate medium for holding deep discussions.

Overall, the relatives personally felt well prepared for the end of life of the palliative resident. However, the statements also revealed the intense stress that the situation caused in some cases.

*“I actually feel well prepared*. *My wish is that he falls asleep in peace*. *I am also thinking about myself*. *I just sit here and do everything for my husband*. *What do I get out of life*?”

*(ZM_1)* ^91^

*“Hmm*, *it might sound strange*, *but I would be pleased for him if he were to survive*, *you know*? *But I would also not fall into a deep hole*, *certainly not*. *Seeing him sick*, *makes me sick*, *you know*? *It makes me sick*, *and then I also cry a lot*.”

*(ZM_2)* ^92^

In general, relatives asked, among other things, for shorter waiting times when a resident buzzes, someone to look after the residents more often, more activity programmes for residents and, for bedridden people, the opportunity to go outside (to a balcony or garden), particularly for those residents without relatives.

## Discussion

The T1 results revealed shifts as well as congruities and discrepancies in problem areas and challenges concerning hospice and palliative care during the COVID-19 pandemic relative to baseline. These shifts were perceived differently by the different perspectives. An overview of the main findings within the interviews from all three perspectives at baseline (T0) and T1, which were described above, can be found under S2 File.

### Uncertainties and need for information (during COVID-19)

At T1 as well as T0, the identification of the need for palliative care continued to be based on the empirical values of employees, without the use of validated screening instruments. However, the respondents noted that the identification improved. In contrast to T0, uncertainties defining the palliative phase were only described by managers in relation to employees. A possible explanation for this could be participation in the project. A greater focus on the topic of “palliative and hospice care” in the involved nursing homes may have led to greater awareness of the indications of palliative care situations among employees and a closer look at their uncertainties, especially those employees who reflected on their uncertainties during the T0 interviews.

To reduce uncertainties, employees indicated the need for more information about palliative structures and processes in the nursing homes. Increased information could also meet the needs of residents and relatives, who stated in the interviews that they needed more information about the end of life and dying culture in their respective nursing homes as well as earlier clarification of the procedure and organisational options in the last phase of life.

An implementation of guidelines or recommendations on palliative care during the pandemic, which could have helped to provide more structure and clarification of processes during the pandemic and thus to reduce uncertainties, was not mentioned by employees or managers or by residents or relatives. Within a recent scoping review [46], five guidelines and recommendations were identified for Germany [47–51], four of which could be applied to palliative care within nursing homes [48–51]. Because there is no indication that these guidelines and recommendations have been used within the nursing homes in this study, it would be interesting to investigate in future research to what degree the guidelines have been applied in other facilities and with what results.

### Communication, contact and potential of digitalisation

The importance of the topic “communication and contact” increased considerably during the COVID-19 pandemic. During the period of visitor restrictions, employees often also acted as “go-betweens”, as they were the only personal contact for (palliative) residents. In addition, employees were often also the only way for relatives to establish contact with residents and obtain information about their condition.

Due to contact restrictions, important parts of end-of-life care were missing. Although the majority of employees stated that communication with relatives during this period was impaired, problematic in some cases and time-consuming, one person described that they found the lack of visits to be a positive. The reason given was fewer interruptions due to the need for meetings and information by relatives, which provided a greater sense of calmness and more time to care for residents. However, this attitude needs to be questioned critically. According to the principals of palliative care, relatives play an important role in the multiprofessional palliative care team, just as caregivers, doctors or other professions do. Hence, relatives should be considered partners pursuing a common goal of supporting palliative residents according to their wishes in their last stage of life and less as an additional workload. Relatives may even help to reduce the workload of employees by taking over minor care tasks or simply by being present.

In contrast to employees, relatives were more satisfied with communication with employees at T1 than at T0 due to the reduced opportunities for communication during the pandemic and the associated lower expectations. However, at T1, relatives found it more difficult to deal with conflicts with employees, as they were more reliant on employees due to their own, reduced opportunities for contact with palliative residents. This reliance led to greater fear that addressing conflicts would have a negative impact on the care relationship, potentially preventing relatives from expressing their own or the perceived needs of the resident to employees, which may have fostered dissatisfaction and frustration. In some cases, employees reported escalating situations in dealing with relatives due to a lack of understanding of the protection measures. Earlier, open communication on both sides could help in such cases, as this would prevent negative emotions from building up and escalating.

However, relatives described the feeling that employees responded better to their needs at T1, which was a positive development. Although the needs of relatives were not considered by employees at T0, at T1, several relatives received feedback indicating that they should take more time to look out for themselves and that they could rely on the staff. Employees also reported a closer relationship with residents as well as relatives during the pandemic. One reason for this may be that employees were aware that they were the only social contact for residents during the time of visitor restrictions. Thus, residents directly relied on them for contact with people outside the nursing home. Conversely, employees were also the only way for relatives to contact their palliative residents. However, this was not described from the perspective of the residents. The majority stated that their relationship with employees remained unchanged during the pandemic.

Residents wished for more contact with employees to receive information. Although some employees described that they paid more frequent visits to residents and exhibited a greater presence, there seemed to be little if any information exchange concerning the needs of residents. In the future, additional research is required to determine the precise structure of contact between residents and employees. How much time is actually spent on conversations, and what level of presence is involved? How are the conversations structured, and what is the content? Do the questions concern the general well-being, or are more in-depth conversations also held? Is the attitude shown towards residents one that allows questions to be asked?

Digital media became significantly more important for communication for all three groups during the pandemic. The telephone was the most commonly used means of communication. Interestingly, although the lack of contact between residents and relatives was described as stressful, in certain cases, all three groups preferred no contact compared with (video) calls. Relatives reported confusion among residents, and employees stated that, in some cases, video/telephone contact triggered negative feelings in residents or that they responded with great sadness. This poses the question of whether, in these cases, the opinion of the resident concerned was in fact considered. This is particularly concerning, in terms of self-determination, if the residents themselves wanted video/telephone contact despite any associated negative feelings but were denied video/telephone contact due to the opinions of others. The residents overwhelmingly stated that they maintained contact, usually through an increase in phone calls.

In other nursing homes, the telephone was also the most frequently used digital communication technology during the pandemic. In a cross-sectional survey of 824 nursing homes in Germany, 86.4% answered that they used the telephone for communication with external collaboration partners, followed by e-mail (73.8%) [52]. For communication with relatives, 90.9% and 77.9% answered that they used the telephone and e-mail, respectively [52]. The surveyed nursing homes also used digital communication options to get in touch with residents themselves. A total of 54.4% of the nursing homes used the telephone for this purpose; e-mail was used less commonly (19.2%) [52]. Only 13% of the nursing homes used videoconferencing to communicate with external collaboration partners [52]. Forty percent answered that they used videoconferencing with relatives, and 25% used it for communication with residents [52]. The majority of nursing homes within the study (75.9%) created opportunities for residents during the pandemic to use digital communication technologies for social contact with friends, relatives, or others [52]. For this, many nursing homes have purchased tablets or smartphones and installed programs such as Skype. Some nursing homes had to set up Wi-Fi for their residents so that these digital technologies could be used at all [52].

However, this situation does not necessarily apply to palliative residents and within palliative care. Although more digitalisation takes place in palliative care in general and the COVID-19 pandemic accelerated digitalisation in many sectors, there is insufficient research about digitalisation within palliative care in nursing homes. In a qualitative study, health care professionals of specialised outpatient palliative care, specialised inpatient palliative care, hospice care and oncology wards were interviewed about digital technologies in routine palliative care delivery [53]. The three main functions of digital technology use in palliative care were to coordinate work processes and care delivery, patient-centred care and communication [53]. For this, multiple digital devices were used, including smartphones, computers, fax machines, electronic health records, messenger services and video conferencing [53]. However, the participants saw the most potential in digital technologies when they discreetly supported palliative care as an adjunct to face-to-face contact because palliative care requires human connection that cannot be replaced by technology [53].

With the exception of gathering information about how employees, residents and relatives assured communication and contact during the pandemic, this study did not survey digitalisation or telemedicine within palliative care in nursing homes. However, as it might be beneficial for supporting administrative and organisational activities, employees also reported negative effects of technology use within the interviews due to cognitive limitations of the residents and a low generation-related affinity for technology. Nevertheless, palliative care in nursing homes has the potential to benefit from digital technologies. Even if its use may be limited within direct resident interaction and care, adequate palliative care requires collaborations between different professions and disciplines. Digital technologies may contribute to improving administrative and organisational activities as well as communication within an interdisciplinary palliative care team. Further research could provide insights into this.

### Burdens and need for cooperation and funding

Although the majority of residents surveyed at T1 indicated that they had endured the pandemic quite composedly, relatives perceived feelings of intense stress among palliative residents, the cause of which was primarily attributed to loneliness. Employees also described depressive changes in residents brought about by the pandemic. The discrepancy between the perceptions may be due to the time difference between the interviews and the direct lockdown (cf. limitations), which may be associated with different memories, forgetfulness or the repression of stressful experiences. The general contentment of residents and the attitude of being satisfied with little and not complaining or not wanting to be a burden on anyone may also be a reason for the different views. Among relatives and employees, the perception may have been negatively reinforced by their own stress and the guilty conscience of not being able to be there for residents.

In the interviews, both employees and relatives reported feeling more stressed compared with T0. The majority of relatives found the time during the visitor restrictions in the nursing homes to be stressful. However, some relatives also described the imposed visitor restrictions as a relief, as they allowed the relative to stop making the, in some cases, energy-sapping daily visits without having to blame themselves.

Employees as well as relatives perceived a lack of time among the staff, which was described as the greatest stress factor at both survey times. Both groups also agreed that the situation deteriorated at T1 as a result of the pandemic.

A dramatic rise in personal stress for employees was caused in particular by the combination of additional tasks due to staff absenteeism as a result of the pandemic, with the consequence of no longer being able to meet their standard of resident care together with the concern of infecting their social group and/or residents with the SARS-CoV-2 virus. The fine balance that already existed between time resources and palliative care seemed to be squeezed even further during the COVID-19 pandemic.

There is no question that supporting and caring for dying people in nursing homes under the given circumstances can be extremely challenging. Even with the goal of changing these conditions in the long-term, constantly referring to a lack of resources does not help the current situation. This requires a change in attitude among employees, which may cause an entirely different outcome over the same amount of time. In addition, an existing hospice and palliative network is extremely important. Nursing home employees cannot provide support and care for residents in the last phase of life alone—nor do they need to. However, it is employees’ responsibility to involve appropriate partners where they are needed. This requires a greater focus on the need to expand and establish palliative collaborations. In crises, such as the COVID-19 pandemic, there also needs to be considered to what extent partners can be involved in terms of the quality of palliative care despite the risks.

For palliative patients suffering from advanced and progressive diseases with limited life expectancy, complex symptoms and needs, the German statutory health insurance covers specialised outpatient palliative care (SAPV) [54]. SAPV is provided by multidisciplinary and multiprofessional teams specialised in palliative care at the patient’s residence, which also includes application in nursing homes [54] and thus has the potential to support employees and ensure a high quality of care for palliative patients.

However, studies imply that SAPV is not used to the full extent in nursing homes. Within a survey of nursing homes, nearly two thirds (65.5%) answered that residents should be enrolled in SAPV more frequently [6]. In another study, only 12.8% of the nursing homes (n = 361) answered that they used SAPV daily or weekly, and more than a quarter (26%) answered that they never used SAPV [55]. Additionally, the sample within this study used SAPV only occasionally [43]. During the pandemic, SAPV teams described greater problems in collaborations (e.g., nursing homes) within a nationwide online survey [56]. Furthermore, the general avoidance of contacts, compliance with hygiene regulations for personal contacts, and pandemic-related fears, both within the team and among relatives and patients, made SAPV more difficult in nursing homes [56].

Nevertheless, the integration of SAPV requires trained and sensitised employees in nursing homes who have palliative care-specific expertise, know the structures of palliative care, can identify the need for SAPV and can initiate SAPV support. As employees and managers described uncertainties regarding the palliative phase and the need for more information about palliative structures and processes, it is doubtful that these conditions were met, at least within this study. To improve this situation and integrate SAPV more reliably when needed, a greater focus should be placed on providing palliative care expertise in nursing homes as well as clarifying structures and collaborations within palliative care. Furthermore, SAPV teams should be part of every nursing home’s collaboration partners and be more integrated into already existing palliative networks by legal guidelines.

With the introduction of SAPV in 2007, there is an entitlement from health insurance for palliative residents with particularly complex care needs, yet there is no refinancing of general palliative care, which accounts for approximately 90% of all palliative cases [8, 16, 17]. By law, care explicitly includes end-of-life care (§ 28 Abs. 4 SGB XI). However, high quantitative work demands due to time and personnel resources allow insufficient time for adequate end-of-life care in the hectic daily work routine in nursing homes [47], which often requires greater personnel and time resources. This lack of resources can not only lead to psychosocial stress for employees but can also negatively impact the quality and safety of care for palliative residents. In a nationwide representative survey on working conditions, 42% of 598 nursing home employees often or very often cut back on the quality of their work to cope with their workload [57]. As most considered palliative care to be a particularly important activity, reductions in the quality of care often had an impact on their sense of stress [55, 57]. Thus, 35% of employees who stated that they cut back on the quality of their work often or very often reported negative psychological stress [57]. As a result, the funding situation for palliative care exacerbates the already strained working and staffing conditions in nursing homes, which was described as even more intense during the pandemic by all three perspectives within this study. Hence, there is an urgent need for health policy action to establish a funding basis for palliative care in nursing homes.

### Requests in the last phase of life and need for palliative expertise

The successful fulfilment of the requests of palliative residents was perceived differently by the three groups. While employees reported that they were largely able to fulfil requests, except requests for visits during visitor restrictions, relatives indicated that minor, day-to-day requests could not be reliably fulfilled. Employees focused on larger, one-off concerns, according to the relatives. At T1 as well as T0, the residents themselves described that, in some cases, they did not even bother making requests in the first place, and where minor requests were made, they remained largely unfulfilled. Even though these kinds of “minor” requests may be considered insignificant or remain unnoticed by employees, they are very important for residents and their relatives and have a material influence on everyday life and satisfaction. However, for example, taking telephones from residents (see relatives, results chapter) because not all employees are able to place the handset away in the evening is a serious encroachment ono the right to self-determination and demonstrates that, in some cases, the implementation of requests and attempts to establish a certain level of autonomy fail due to a lack of organisational solutions in the nursing homes.

Despite the direct threat from the pandemic, very few conversations were held on specific requests and expectations in the last phase of life. At T1, this may have been due to the limited time of employees and the fact that, for relatives, discussions concerning the last phase of life required personal communication. With regard to expressing requests, in some cases, residents were even more unassuming and reticent at T1 given their awareness of the considerable pandemic-related stress on employees.

While requests by relatives, such as for shorter waiting times for palliative residents when expressing concerns, increased during the pandemic, they were also already present at T0. Employees did not address the extent to which the concerns of relatives could be incorporated into resident care. They primarily described an increased need for communication by relatives, which required a greater amount of time. Given the obstacles described by relatives concerning the fear of conflicts that may negatively affect the relationship with employees, it is questionable whether relatives felt comfortable enough to express requests and, if so, whether employees considered these and ascribed any importance to them.

At both T0 and T1, relatives of palliative residents made reference to the extent to which the care and adherence to arrangements depended on the specific employee. However, this poses the question of whether a good level of hospice and palliative care should be ensured irrespective of the employee.

What needs be noted regarding this topic, however, is that palliative-specific expertise of employees is expected and needed, yet the content of palliative care is not uniformly regulated in the basic training of nurses [58]. As a result, employees have different levels of understanding and knowledge about palliative care, and only one in five nurses in Germany has palliative care expertise due to specialised training [47]. Since January 2020, a new nursing training program has been introduced in Germany that combines the training programs of paediatric nursing, geriatric nursing and general nursing in whose curricula a module regarding accompanying people in critical life situations and in the final phase of life is recommended, but content and scope are not obligatory [59]. Hence, to pose the question of whether a good level of hospice and palliative care should be ensured irrespective of certain employees, it is first necessary to ensure that staff have a certain level of basic palliative competence. The German Society for Palliative Medicine recommends integrating basic competencies for general palliative care in hospitals, inpatient geriatric care, rehabilitation care and outpatient care into the training curricula of nursing staff [60]; however, due to the recommendatory character of framework curricula, implementation—even if integrated into the curricula—is not obligatory.

Despite the scope of a uniform level of basic palliative competencies in nursing training, what nursing homes can do to help is clarify the mutual expectations of employees, residents and relatives with regard to what palliative residents and their relatives can expect from all employees, where employees can be more flexible in their responses, and how “minor wishes” can be fulfilled. Again, providing more information about palliative care, organisation, structures and processes to all three perspectives could help in this matter.

### Limitations

The aim of the present study was to gain a multiperspective insight into the extent to which hospice and palliative care in nursing homes has changed due to the COVID-19 pandemic. However, the scope of the results is limited. First, the interviews could only be held in two nursing homes and with only a relatively small number of interviewees, which may limit the external validity and should be taken into account when transferring the results. Interviewees were selected unsystematically by project staff within the nursing homes, meaning that only certain people were given access to the study, which thus may include a certain bias. Second, it was not possible in every case to interview the same people at T0 and T1, as some were no longer available or had died. Furthermore, the number of interviewed residents and relatives varied from T0 to T1, as the pandemic has made recruiting a corresponding number of interview partners in these two groups impossible. Because insights from these two perspectives are scarce and difficult to survey, a lower number of interviewees at T1 was nevertheless considered valuable. Ultimately, a period for the interviews at T1 was selected in which personal meetings in the nursing homes had only recently become possible. The interviewees reported on some aspects (e.g., at the time of the ban on visitors) from memory, which may lead to distortions. Furthermore, it must be noted that both nursing homes are located in North Rhine-Westphalia, Germany, and the protection rules during the pandemic differed across the federal states. Therefore, perceptions of changes within hospice and palliative care in nursing homes during the COVID-19 pandemic may differ in other federal states and should be considered when transferring the results. To expand insights, further research is needed within this field. Finally, variation between the nursing homes was sought to include a broad base of viewpoints. However, subanalyses of different criteria, e.g., differences between rural and urban nursing home locations or differences between the nursing homes, were not covered within this article.

## Conclusions and perspectives

The results reveal changes, challenges and problem areas concerning hospice and palliative care in nursing homes during the COVID-19 pandemic from the perspectives of employees, residents and relatives. To improve the situation of hospice and palliative care in nursing homes, not only during pandemic times, the following conclusions can be drawn.

### Practical implications

- A certain level of expertise of health care professionals in hospice and palliative care needs to be assured. In addition to more awareness, more hospice and palliative-specific training of all professions (administration, housekeeping, social services, nursing staff and assistants, management, doctors, rescue services, etc.) is needed, as most residents spend their end of life in nursing homes and it is insufficient to call in specialised palliative care from time to time.
- The majority of nurses in nursing homes should have a palliative care certificate.
- Expectations and possibilities between staff, residents and relatives should be clarified, and a greater understanding of the different needs should be created. This is only possible if sufficient opportunities for discussion and information are available.
- Palliative cooperation should be expanded. To provide the best possible care for each individual person in the last phase of life, multiple interdisciplinary professions should be part of palliative care teams in nursing homes and be reliably integrated when needed. The use of digital technologies and telemedicine may help to support this (e.g., SAPV-video consultation).

### Political/structural recommendations

- Palliative care and its scope should be mandatorily integrated into nursing education and not just named in the curriculum as recommended.
- A funding basis for general palliative care in nursing homes should be established, e.g., by considering hospice and palliative care in budget negotiations.
- There should be a legal obligation for nursing homes to cooperate with hospice and palliative care providers (e.g., SAPV).
- Nursing homes should be integrated into the hospice and palliative care networks in the area.
- Policy measures should focus on improving standard palliative care rather than expanding increasingly specialised palliative care, for which only a small proportion of the population has the need.

### Research gaps

- There is no evaluation of the integration of COVID-19 guidelines and recommendations in nursing facilities and their effects.
- Subanalyses, e.g., between facilities or on differences between rural and urban areas, are still pending.
- Analyses of the situation in other states and the transferability of the results would provide broader insights into this research field.
- Further research is needed on how digital technologies and telemedicine may be used to improve palliative care (e.g., in the care context of digital diaries on symptoms or video consultations as well as in administration, organisation or communication between palliative care team members), especially in nursing homes.

## Supporting information

S1 File Original interview quotes (German language). (PDF)

S2 File Main findings from all perspectives at T0 and T1. (PDF)
