# Supplementary material for: Effects of the COVID-19 pandemic on hospice and palliative care in nursing homes—A qualitative study from a multiperspective view
Source: PLoS One. 2023 Oct 5;18(10):e0286875. doi: 10.1371/journal.pone.0286875 (PMC10553271; doi:10.1371/journal.pone.0286875)
Supplement: S1 File — (PDF) [file pone.0286875.s001.pdf]

S1. Original quotes in German

Employees

[1] „Für mich ist nicht ganz klar, wann wir jetzt tatsächlich aktiv werden. Klar, wenn es eine gesundheitliche Veränderung gibt und wir sehen „oh, da macht sich einer auf die Reise“ also das finde ich müsste nochmal aufgestellt werden. Das würde mir Sicherheit geben. […] Das ist eigentlich noch nicht geregelt, finde ich, ja? Das macht mir so ein bisschen die Unsicherheit, dass ich mir das sozusagen selbst suchen muss.“ (MA_2)

[2] „Manchmal liegen wir aber auch einfach falsch. […] Da es ist dann manchmal so ein bisschen Versuch und Irrtum.“ (LF_3)

[3] „Manchmal glaub ich, dass ist vielleicht auch weil ich noch sehr jung bin. Vielleicht kommt das mit der Erfahrung, also ich hoffe zumindest. Aber wie gesagt, ich hoffe, vielleicht lerne ich das auch, wenn ich die palliative Versorgung da mache, die Weiterbildung. Vielleicht lerne ich das. Ich hoffe.“ (MF_3)

[4] „Also, was ich wirklich bemängle ist, wie wir mit Ärzten aufgestellt sind, die ärztliche Unterstützung im Palliativbereich. Das würde ich mir besser wünschen. Allein schon für so Fälle wie, Fallbesprechungen, ethische Fallbesprechungen und so etwas in dieser Richtung, Konsiliare. Hab ich eigentlich kaum mitgekriegt, dass man da wirklich verlässliche Unterstützung seitens der Ärzte hat und das finde ich auch manchmal dringend notwendig.“ (MF_2)

[5] „Bei den Ärzten, teilweise sehen sie die Dringlichkeit nicht so, so nach dem Motto: „Ja, ich komme Morgen, Übermorgen.“ „Ne, jetzt. Heute. Morgen ist er vielleicht schon tot.“ So, oder wenn man sagt: „Ja, der ist in der palliativen Phase aber noch nicht so weit, dass wir jetzt irgendwie Tavor oder Morphium aufschreiben. Zur Not haben Sie ja etwas da.“ „Nein, das ist Diebstahl. Wir dürfen nicht von einem anderen Bewohner etwas wegnehmen.“ (LA_4)

[6] „[…] aber die Menschen, die in die Sterbephase geraten, die möchten bekannte Gesichter. Also wenn da jemand vom Hospiz kommt, der ist fremd. Also man weiß ja noch nicht mal ist da die Sympathie da, „der kennt mich gar nicht“. Also das Angebot wird tatsächlich wenig wahrgenommen […].“ (MA_4)

[7] „Ich weiß, dass bestimmte Prozesse stattfinden. Ich bezweifele, dass die wirklich immer so geregelt sind, dass die geregelt ablaufen und ich weiß für mich zu sagen, dass wenn diese Regelungen existieren sollten, ich sie bisher noch nicht so verinnerlichen konnte oder sie noch nicht an mich herangetragen wurden. Ich glaub das geht vielen so. […] Mein Eindruck ist, auch aufgrund meiner eigenen Unsicherheit, irgendwie so, ich hab so meine Zweifel ob mir das immer so bewusst wird, wie es vielleicht abzulaufen hat.“ (MA_2)

[8] „[…] man hat nie so vorher das mal so durchgenommen. Wie verhält man sich? Wie ist hier so der Ablauf? Ich weiß noch, als ich hier angefangen habe, da war jemand in der Phase und ist dann während der Übergabe verstorben und ich wusste da gar nicht, was ich machen sollte.“ (MF_5)

[9] „Ja, dann fällt die komplette Grundpflege oder Dusche halt eben weg. […] Weil derjenige sagt: „Zu mehr bin ich nicht in der Lage und mehr wünsche ich nicht. Ich bin doch sauber.“ Das sind so Sachen, wo man dann auch wirklich gucken muss und sagen muss: „Ja, das ist okay.“ Das muss aber von den Mitarbeitern auch wirklich reinkommen und das muss umgesetzt werden. Das wird aber da merken Sie, da haben die Schwierigkeiten mit. Der eine mehr, der andere weniger.“ (LF_2)

[10] „So, ich kann jetzt für mich selber sagen, ich gehe nicht immer mit einem guten Gefühl nachhause und denke, all meine Bewohner sind jetzt über die Nacht gut versorgt, nein, tue ich nicht und es wäre auch, es wäre auch wünschenswert.“ (LA_3)

[11] „Ich habe da manchmal so Schwierigkeiten, wenn die Menschen, das sag ich ganz böse, aus dem Krankenhaus ausgeschüttet werden. Zwei, drei Tage verbleiben, wo ich mir denke: „Wo bleibt die Menschlichkeit? Wo bleibt der Mensch?“ […] Oft kommen sie dann ohne Medikamente, wo ich dann zu viel kriege. Dann geben sie das nicht mit oder haben es vergessen. […] Dann ist man echt verzweifelt.“ (LA_2)

S1. Original quotes in German

[12] „An sich die Belastung, hier mit Leiden und Tod konfrontiert zu werden. […] Es ist nicht leicht und es auch nicht leicht in einem Umfeld zu arbeiten, wo man eigentlich sehr, sehr stark und fortwährend mit Leid, Unzufriedenheit, Ängsten und den ganzen düsteren Gedanken zu tun hat. Da dem immer etwas Positives entgegenstellen zu wollen geht manchmal an die Kraft, ne. Und eben auch das Bewusstsein, dass man das überhaupt nicht immer schaffen kann. Aber es ist eine sehr kraftraubende Tätigkeit.“ (MF_2)

[13] „[…] dass man sich nicht einfach ans Bett setzen kann und eine Hand nehmen ne, oder auch mal über das Gesicht streicheln kann, […] Es ist ein anderes Gefühl, es mit Handschuhen zu tun und Kittelchen und die Person hat keinen Hautkontakt.“ (ME_6)

[14] „Dat heißt, die erkennen gar nicht, ob dann Männlein, Weiblein reinkommt, […]. Man hat n Faceshield auf, ne Brille, n Haarnetz, n Kittel und ne Maske. Und die Kommunikation ist äh fast kaum möglich. Wenn man da jemand hat, der schlecht hört, äh ist schon ganz vorbei. Schlecht sieht auch und demenziell veränderte verstehen dann gar nicht mehr, was los ist.“ (ME_7)

[15] „Also bis man die Ärzte erstmal am Telefon hatte, dass wir dann teilweise wirklich Aufwand hatten, hier Faxe zu schreiben und, ne. „Wir brauchen Sie. Machen Sie mal. Rufen Sie mal zurück.“ Und das war ganz schlimm. […] Ich fand es extremer, weil vorher hat man sie eigentlich noch relativ oft ans Telefon bekommen. Aber seit Pandemiebeginn ist das wirklich ein No-Go. Ganz schlimm finde ich das.“ (MM_6)

[16] „Also ist natürlich das eine oder andere an Qualität vielleicht für palliativen Bewohner auch natürlich dann äh auf der Strecke geblieben. Man will ja alle bedienen und alle zufrieden stellen, ne.“ (MM_1)

[17] „Die waren dann schon ein stückweit Familienersatz, was sie ja eigentlich gar nicht sein wollen und sein dürfen und sein können. […] Aber in solchen besonderen Situationen haben wir dann natürlich auch ähm ein bisschen mehr zugelassen. Auch wir Leitungskräfte haben mehr Nähe toleriert, die wir zulassen zwischen unseren Mitarbeitern und den Bewohnern.“ (ME_1)

[18] „Und vor allen Dingen auch dieses „Ja, das ist schön, dass ihr jetzt Fotos schickt. Damit kann der Bewohner jetzt gerade nichts anfangen.“ Oder ihr schickt jetzt ein Video und der Bewohner hat das Gefühl, ihr seid live gegenüber und spricht mit euch. Kann das gar nicht richtig realisieren. Also dann hat man, manchmal hatte man auch das Gefühl, das bewirkt jetzt eigentlich nichts Positives, sondern eher was Negatives. Wir hatten ja auch drei Bewohner, die ähm haben immer sofort geheult, sobald sie Ihre Tochter ihren Sohn da am Tablet gesehen haben oder auch am am Ohr hatten. Muss man sagen „Das geht jetzt im Moment nicht. Wir müssen jetzt hier abbrechen.“ (ME_5)

[19] „Oh ich glaube, die werden den [Bewohner*innenwünschen] schon gerecht. Und wenn nicht, versuchen sie es und machen alles möglich, dass Sie, dass Sie diesen den Wünschen Bedürfnissen gerecht werden. Auf jeden Fall. Ich glaube, dass das funktioniert ganz gut. Ja.“ (ME_2)

[20] „Einige, die viel Verständnis haben, einige, die gar kein Verständnis haben, die vor der Einrichtung äh gebrüllt, geweint haben, uns die Schuld gegeben haben, dass man die Mutter nicht mehr sehen konnte. Ähm dann uns mit Polizei und weiß nicht was gedroht haben.“ (ME_1)

[21] „Weil die hatten ja dann auch Gesprächsbedarf und die Angehörigen riefen an und die musste man ja auch informieren und beruhigen und solche Sachen. […] Und das war schon mit Mehrarbeit verbunden, auf jeden Fall.“ (MM_1)

[22] „Am Anfang war es schon sehr große Verunsicherung, […] Sie haben keine Ahnung von dieser verdammten Erkrankung, von diesem Virus. Sie haben keine Ahnung, wie sich das verhält. Sie kommen sich vor, wie in nem, na wenn Sie zum Dienst fahren, Lockdown. Die Straßen sind leer und dann kommen Sie hier angetuckert. Dann geht hier eine (Schhh) Schleuse auf und dann geht (Schhh) und dann gehen Sie und packen.“ (MM_1)

S1. Original quotes in German

[23] „[…] da hat man ja auch Angst, vielleicht die palliativen Bewohner noch mehr irgendwie zu gefährden oder auch anzustecken. […] Also ich denke, dass man sich da schon kürzer auch aufgehalten hat, als man vielleicht auch mal manchmal müsste." (ME_3)

[24] „Und nochmal ins Zimmer gehen und äh da ist jetzt die nächsten drei Stunden keiner im Zimmer. Dat heißt ich muss da 4, 5, 6 Mal reingehen, einfach mal gucken, weil et ist niemand präsent. Das wird, das hat zugenommen, ja." (ME_7)

[25] „Eher unsicher. […] Ja ich glaube, dass sie selber Angst haben, jemanden als palliativ einzuschätzen oder äh das nicht richtig einschätzen können. […] Gestern äh Situationen bin ich schon dazu gekommen „Was? Wie schätzt ihr es denn ein?" Und ich frag dann auch und aber die sind einfach total unsicher." (ME_2)

[26] „[…] dass man auch darüber aufklärt, was gibt es Neues in der palliativen Versorgung, ne. […] oder was im Haus gerade ist. Wir können zwar immer in Gesprächen dabei sein, aber ob dann immer alles so kommuniziert wird, woran man dann vielleicht im Nachhinein denkt: „Und ach, hättest du nochmal." Würde ich so einen palliativen Nachmittag, wo man einmal darüber aufklärt, auch über die Bewohner, schon gut finden." (ME_3)

[27] „Wir haben jetzt viel deutlicher klar, wer ist für, wer ist in einer palliativen Versorgungssituation und äh wie ist die Situation gerade?" (ME_1)

[28] „Also Pflege hat immer, Pflege und Betreuung hat immer Zeitdruck. […] Aber na klar, es hat sie natürlich nochmal verschärft. […] Allein schon, wie oft die Kollegen dann Hände desinfizieren oder waschen, ähm wenn ich das hochrechne, alleine darüber komme ich ja schon auf einen Wert, der mir in der effektiven Versorgung fehlt." (ME_1)

[29] „Also wir waren über Fußpflege, Frisör, Seelsorge, ja Familie einfach mal zum in Arm nehmen, zuhören. […] Und, also es hat schon Management gefordert." (MM_4)

[30] „Wenn man unter den Bedingungen arbeitet, muss man nahezu täglich priorisieren. Was hat heute absoluten Vorrang? Was muss ich erledigen? Was muss geschafft werden im wahrsten Sinne des Wortes und welche Punkte kann ich schieben?" (ME_1)

[31] „[…] ich kann mich ähm nicht hundert Prozentig ähm bei dem Bewohner hinsetzen und abschalten und nur für den Bewohner da sein. Dat schaff ich persönlich nicht. Mit dem Herzen vielleicht, aber mit dem Kopf nicht." (ME_6)

[32] „Und da bin ich gerade auch noch ganz ratlos, wie wir, wie wir das im Nachgang nach Pandemie, und es muss irgendwann eine Zeit nach Pandemie geben, wie wir das gekittet kriegen, weil die Qualität, wie Menschen zum Teil verstorben sind. In was für einer rasanten Geschwindigkeit äh das war nie dagewesen. Und wer da nicht am Bett gestanden hat in diesen Momenten, der wird es auch nicht glauben. […] Und da sind viele Tränen geflossen. Da gab es Verzweiflung, weil man helfen wollte und einfach nicht konnte, weil […] wir die technisch-medizinischen Voraussetzungen dafür nicht haben" (ME_1)

Residents

[33] „Traurig bin ich dauernd." (BA_3)

[34] „Die fragen immer was woll'n Sie ich sag ich will gar nix ich bin froh wenn ich meinen Sprudel hab meine Luft hab und die Zeitungen und Besuch kriech." (BA_4)

[35] „Eine Bewohnerin bekommt statt Marmelade morgens immer Käse." (BF_5)

[36] „Ja ja, die hat Probleme mit den Zähnen während dem Essen nimmt se denn die Zähne raus und legt se aufn Tisch und die anderen die tut nach dem Essen die Reste raus holen also deswegen am liebsten will ich ganz aufm Zimmer essen." (BF_3)

[37] „Ich hab ich hab sehr viel Kontakt mit Bewohnern." (BA_7)

S1. Original quotes in German

[38] „Ne ist ja keiner da mit dem ich reden kann.(...) „Wie ja warum, oder…“ wird nich gefragt, da haben die auch keine Zeit hier.“ (BF_2)

[39] „Nein et gibt Angebote aber dat is meist für diejenigen die gar nicht mehr so können und die nich mehr so ganz klar denken können ich hab keine Lust mich hinzusetzen äh Mensch ärger dich nicht zuspielen oder so wat.“ (BA_5)

[40] „Ja sicher gibt das Angebote genug ich mach hier nicht alles mit aber vieles mit ne.“ (BF_4)

[41] „Ich möchte einfach aufstehen umkippen und weg sein so wie mein Mann, ja.“ (BA_7)

[42] „Ich seh locker weil wir kommen ja doch nicht drum hin.“ (BF_5)

[43] „Ne, das also eigentlich regeln unsere wir haben das mit unseren Kindern besprochen.“ (BA_1)

[44] „…möchte ganz für mich alleine sterben ich bräuchte keinen dabei... ja, Jesus der führt mich.“ (BF_4)

[45] „Da hab ich noch nichts von gehört [hospizliche Begleitung].“ (BF_7)

[46] „Ja die die äh Verwandten die kommen die dürfen dann nochmal aber sonst wir selbst wir dürfen dann nicht rein in die Zimmer.“ (BA_5)

[47] „Ne, sagt mir nichts. Das hat sich auch so vorbeigeschlichen, ne, vorbeigegangen, nein gar nichts, nein gar nichts, keine Einschränkungen erlebt […] ich bin mal geimpft worden aber was das jetzt war weiß ich nicht nee.“ (BE_1)

[48] „Es war schlimm zwar aber ich hab‘s überstanden ((lacht)) och Angst nicht, ich hatte ja soweit nichts ne (?)“ (BE2)

[49] „Wir sind ja eigentlich sehr geschützt hier, ne? Das ist äh, wenn draußen, wenn die Zahlen da so höher gingen, wir waren ja wie auf einer Insel.“ (BM_4)

[50] „Na das muss ja sein, sonst hört dat ja nie auf.“ (BE_2)

[51] „Man kann ja nicht einfach sagen: „Das mach ich nicht“, wenn man so in Gesellschaft lebt, muss man sich einfügen, ne?“ (BM_4)

[52] „[…] und dann können wir uns damals vor dem Balkon laufen da da können wir uns dann drüber unterhalten und und, meine da äh äh, man muss da auch für Verständnis für haben; die wollen da keine Schwierigkeiten kriegen, die Angehörigen.“ (BE_3)

[53] „[…] wir mussten ja alle für uns bleiben; gar nichts keiner durfte zu mir, ja das war ja dann furchtbar.“ (BE_2)

[54] „Ja ich hab viel Musik gemacht und den äh mit dem Computer oder Fernseher so ich hab mich schon irgendwie die Zeit totgeschlagen. Ja ich hab immer versucht mich zu beschäftigen, irgendwas findet man immer ((lacht)), was man machen kann.“ (BE_3)

[55] „Dass es mir nicht schlechter geht, dass es mir eventuell besser geht oder es soll so bleiben wie's jetzt ist und mehr nicht.“ (BM_3)

[56] „Ich komme mit allen gut zurecht. Wenn mir irgendwas nicht passt, dann sag ich ihnen das, hab Verständnis wenn ich eine Stunde hier liegen muss bevor jemand kommt. Hab mir schon mal einen Kakao gewünscht aber dann gab es Brühe, das sagt meine Frau dann, wenn sie da ist.“ (BM_1)

[57] „Da müsste doch ne ganz andere Situation geschaffen werden, dass da wenigstens jemand ist, den man auch ansprechen kann, denn und und der auch dann sich auskennt mit dem Patienten dann. Dat wird alles so hinten ran geschoben so dat alles Hauptsache der Laden läuft und dat Geld kommt und der Rest ist nicht mehr.“ (BM_3)

[58] „Nein, nein, noch nicht. Schieb ich weg. Ja ich denke nicht da dran oder denk gut, haste schon Mal so‘n Tag wenn dann einer von der Verwandten gestorben ist könntest auch du sein aber der liebe Gott will dich noch nicht, nee, so denk ich dann und dann ist auch alles weg. Ist unangenehm für

mich, ich will da nicht drüber nachdenken, ich denk, wenn du geholt werden sollst, dann passiert das.“ (BE_1)

[59] „Die Zeit muss erstmal kommen und dann weiß die Frau schon was zu tun ist.“ (BM_1)

[60] „Nee, da mach ich mir ehrlich gesagt keine Gedanken, da vertrau ich meinen Kindern.“ (BM_2)

[61] „Wenn‘s mir mal schlechter geht, sind die Kinder dann da, ne, und dann kommen se. Mein Mann, der ist bei mir, der lässt mich nicht alleine ja und die Kinder, die, wie die können, ne, kommen se.“ (BE_1)

[62] „Nee das hab ich noch nicht gesagt, da mein ich auch, das müssten auch die Kinder mitentscheiden ne? Ob ses, nee ich weiß, ich weiß es nicht. Ich glaub das ist auch ne Sache, das kann man gar nicht so planen, das das kommt wie‘s kommt.“ (BM_4)

[63] „Es ist eben nicht mehr so wie‘s vorher war, man muss eben doch ein paar Abstriche machen. Wir haben früher hier so‘n kleinen Gesangverein gehabt, der ist ist nicht mehr. Viele sind ja auch gestorben, die mit dabei war’n. Das ist hier schon ‘n Unterschied.“ (BM_3)

[64] „Ja nur dass die Kinder zu mir hinkonnten kommen, ne? Aber dat war ja nicht, ne? Ging ja nicht.“ (BE_2)

[65] „Das Einzige, was ich noch vermisst hab, das war der persönliche Besuch der der Familie. Sonst, sonst wüsst ich nichts, das ist ja alles hier weitergelaufen.“ (BM_5)

Relatives

[66] „Es ist nicht so dass die Pflegenden aktiv auf mich Zugehörige zukommen und sagen Ihr Vater nimmt jetzt diese Medikamente heute war der Arzt da das und das ist geändert worden und diese und diese Beschwerden hat Ihr Vater jetzt neu also das ist jetzt nicht.“ (ZF_2)

[67] „Es ist ja auch jeden Tag teilweise jeden Tag ne andere Schwester da wie soll man denn da nen Austausch haben (?)“ (ZA_1)

[68] „Die Neurologin die ins Heim kommt die hatte mir noch nicht mal gewährt dass ich einmal da hinkommen kann zum Gespräch wenn man wechselt will ich ja wenigstens wissen wollte ich gerne was über meine Mutter erzählen und die mal kennen lernen und so aber das geht nich.“ (ZF_4)

[69] „Also ich weiß dass eine Therapeutin zu meinem Mann kommt aber was die macht oder ob der jetzt mit gemacht hat oder nicht ähm ne das erfahr ich nicht so.“ (ZA_5)

[70] „Also den Raum, den meine Mutter jetzt hat, da hab ich selbst ein paar Bilder auf gehangen oder so.“ (ZA_2)

[71] „Ich weiß, dass ich da für manche äh anstrengend und nervig bin und die finden das natürlich anstrengend, dass ich mich da so oft einbringe. Das passt denen natürlich nicht.“ (ZA_1)

[72] „Ja, aber ich glaube da ist so eine Struktur, die ist vorgegeben und da hatte ich jetzt nicht das Gefühl, dass ich da irgendwie was miteinbringen könnte.“ (ZA_6)

[73] „Man hat mir gesagt, ich kann sie halt begleiten, wenn es soweit ist aber bisher ist es ja noch nicht soweit. Wie das jetzt so von statten geht und was man dann da machen kann, weiß ich auch nicht. Keine Ahnung.“ (ZA_2)

[74] „Ich denke schon, dass ich da einbezogen werde. Die haben ja meine Handynummer und rufen mich dann sicher an.“ (ZA_4)

[75] „Da haben wir eigentlich nicht so drüber gesprochen, wie sie sich das vorgestellt hat.“ (ZA_2)

[76] „Wir haben uns im Detail nicht darüber unterhalten. Ja klar, Erdbestattung, Feuerbestattung, solche Sachen schon, aber nicht darüber, wie es zu Ende gehen soll.“ (ZF_5)

S1. Original quotes in German

[77] „Es gibt hier definitiv Rituale, aber die kann ich Ihnen nicht so ähm ähm im Einzelnen aufzählen.“ (ZF_2)

[78] „[…] konnte ich jeden immer anrufen. Also ich hatte mich oft gescheut äh anzurufen, dann haben se gesagt, machen Sie keinen Quatsch, Sie können jeden Tag anrufen.“ (ZM_2)

[79] „Normalerweise haben die sich gemeldet, aber er wurde auch ins Krankenhaus gebracht, ohne mich vorher anzurufen, ne? Er wurde dann weggebracht und wir wussten überhaupt nicht wohin. Wir haben nicht herausgefunden, wo er war. Ich sach: „Bleibt der jetzt da?“ „Ja das wissen wir auch nicht.“ Irgendwann warn die dann, es war schon ganz spät abends, zehn, elf, „Er ist jetzt wieder da“ sachte die Nachtschwester. Rausgekommen ist das erst, als ich plötzlich nen Bericht kriege, aber nach Wochen.“ (ZM_3)

[80] „Am Anfang ja, „Sie müssen auch auf sich achten, verlassen Sie sich auf uns“. Das ist sehr personenabhängig.“ (ZE_4)

[81] „Ja die schimpfen oft mit mir und sagen, ich sollte mich mal erholen oder sollte mal an mich selber denken, ich sollte nicht immer parat stehen, ne? Doch, das schon.“ (ZM_2)

[82] „Die Einsamkeit war für meinen Mann das Schlimmste. Er hat viel geweint.“ (ZM_1)

[83] „[…] aber es war schon schlimm, also oft sehr schlimm […]. Wir telefonieren auch jeden Morgen und dann ja „Ach komm doch“. Ich sag „Es geht nicht, ich darf nicht ins Heim.“ (ZE_2)

[84] „Einmal fand ich‘s ganz schlimm, da wollt ich zu ihr nachmittags und in dem Moment ähm wurden die Türen dann geschlossen. Da hab ich dann was abgegeben und sie stand von innen hinter so ner Glastür und ich von außen und das war also so ein ganz schlimmer Moment für mich und auch für sie vor allen Dingen.“ (ZE_2)

[85] „Ja ich sach mal so die hat das nicht verstanden. Sie hat die ganze Pandemie nicht verstanden, ne? Das ist so „Warum kommst hier rein und trägst so nen Scheiß im Gesicht?“. Ja verstanden hat die das nicht, da konnte man ihr auch erklären, dann hat sie gesagt „Ja kannst du den Scheiß da mal abmachen?“ (ZE_1)

[86] „Dann müssen Sie ihm den Hörer halt wegnehmen.“ (ZM_3)

[87] „Konflikte werden abgewälzt, sich Fehler eingestehen ist ja sehr schwer, aber wir sind ja auch auf die angewiesen, da muss man schon vorsichtig sein.“ (ZM_1)

[88] „Ich war schon mehrfach bei der Heimleitung und habe auch noch dort angerufen, aber es hat sich eigentlich groß nichts geändert.“ (ZE_2)

[89] „Ja ich würde mir mehr Unterhaltung für die alten Leutchen wünschen, die dann nur eben in ihrem Zimmer sitzen und weil eben nichts gemacht wurde und das wünsch ich mir von ganzem Herzen, aber es geht halt nicht, weil kein Personal da ist, ne?“ (ZE_2)

[90] „Ich sprech mit ihr ungern darüber, weil sie dann weint. Wenn sie einen guten Tag hat könnte man mit ihr darüber sprechen, aber ich habe selbst Angst davor. Das ist so endgültig (weint).“ (ZE_4)

[91] „Ich fühle mich eigentlich gut vorbereitet. Ich wünsche mir, dass er in Ruhe einschlafen kann. Ich denke dabei auch an mich. Ich sitze nur noch da und mache alles für meinen Mann. Was habe ich noch vom Leben?“ (ZM_1)

[92] „Also ich würde ähm, das hört sich vielleicht jetzt komisch an, aber ich würde ihm gönnen, er würde es überstehen, ne? Und ich würde auch nicht in ein tiefes Loch fallen, ganz bestimmt nicht. Mich macht es krank, wenn er krank ist, ne? Das macht mich krank, aber ähm dann wein ich auch oft.“ (ZM_2)
