# Supplementary material for: Effects of the COVID-19 pandemic on hospice and palliative care in nursing homes—A qualitative study from a multiperspective view
Source: PLoS One. 2023 Oct 5;18(10):e0286875. doi: 10.1371/journal.pone.0286875 (PMC10553271; doi:10.1371/journal.pone.0286875)
Supplement: S2 File — (PDF) [file pone.0286875.s002.pdf]

S2. Main findings from all perspectives at T0 and T1

Employees

T0	T1
**Identification of the need for palliative care**<br>- Predominantly based on subjective values of employees in their daily routine<br>- No use of assessments<br>- Reliable identification questionable<br>- Problematic for inexperienced employees who expressed uncertainties in this respect	**Identification of the need for palliative care**<br>- Identification predominantly based on subjective values of employees in their daily routine<br>- Uncertainties regarding the identification of the need of palliative care, the assessment of palliative symptoms and the process and communication channels<br>- Protective equipment restricted communication with residents
**Collaboration with cooperation partners**<br>- Desire for greater availability of medical staff and stronger on-site presence<br>- Different perception of cooperation partners and nursing home staff with regard to the urgency of situations in the palliative phase<br>- Unstructured involvement of outpatient hospice services based on the perception of employees	**Collaboration with cooperation partners**<br>- Reduced end-of-life care due to visiting restrictions (exceptions in the terminal phase)<br>- Difficulties in reaching cooperation partners, e.g., doctors<br>- Less medical presence on site<br>- Temporary suspension of hospice care and pastoral care by cooperation partners<br>- Communication with cooperation partners and relatives occurred predominantly via telephone
**Uncertainties in the daily work routine**<br>- Uncertainties in palliative care processes due to a lack of structural and organisational specifications<br>- Uncertainties in the coordination of time-critical palliative processes and activities<br>- Uncertainties in dealing with relatives of palliative residents<br>- Uncertainties with respect to when to focus on care and human attention rather than on routine care processes in the palliative phase	**Uncertainties in the daily work routine**<br>- Uncertainties regarding the pandemic situation<br>- Pandemic-related (short-term) adjustments in the facility<br>- Media reporting<br>- Dealing with palliative residents to minimise risk of infection<br>- Dealing with relatives who sometimes had different levels of understanding to the pandemic-related restrictions
**Stress in the context of palliative care**<br>- Sparse time resources for hospice and palliative care as the greatest stress factor<br>- Stress due to guilty conscience<br>- Stress due to relocations of dying people to the nursing home on short notice<br>- Uncertainties in the transition from care processes in the palliative phase as a significant stress factor<br>- Stress due to the absence of resources, such as medication<br>- Stress due to non-specific arrangements and/or unclear medication<br>- Stress due to potential conflict with relatives	**Stress in the context of palliative care**<br>- Hospice and palliative care under protective equipment was perceived as stressful<br>- Low time and personnel resources for palliative care as the greatest stress factor<br>- Pandemic-related additional workload due to testing, protective equipment and compensating for social contacts with unchanged or reduced staff due to quarantines<br>- Compensating for staff shortages by filling in at short notice<br>- Prioritising tasks on a daily basis in order to cope with the workload

- Palliative care was described as exhausting work	- Change from group to individual activities for residents under the same personnel resources<br>- Employees compensating social contacts due to visiting restrictions and reduced activities<br>- More time resources required due to a higher need for information and conversations of residents and relatives<br>- Fears and worries about infecting oneself, one's social environment and/or residents<br>- Sadness and sympathy for palliative residents, having to spend the end of their lives under pandemic conditions<br>- Stress due to the speed of symptom increase and deterioration of the general condition of corona-positive residents<br>- Feeling of helplessness

Residents

T0	T1
**Well-being and care**<br>- Most residents reported feeling stressed and/or sad<br>- Residents indicated that they were largely satisfied and did not have any specific requests. There often was a lack of comprehension of the question, as the possibility of expressing a request was viewed as absurd<br>- If there were requests, they tended not to be expressed ("It won't be met, so I don't ask, that's what I have learned in my life")<br>- If requests were expressed, they tended not to be met<br>- The topic of eating played an important role in the meetings: many were not satisfied with it and/or felt shame due to unseemly behaviour as a result of problems with teeth, shaking hands or impaired eyesight	**Well-being and care**<br>- For the majority of those surveyed, the pandemic was not a defining, negative experience. Most endured it more or less composedly<br>- Despite being vaccinated, three people caught COVID-19 and spent up to three weeks in quarantine. This was endured more or less composedly and without great fear<br>- The ability to be comfortable spending time alone was found to be helpful during the COVID-19 period<br>- Requests concerning care and support tended not to be expressed and, if they were, they were of a more general nature<br>- Whenever minor requests were expressed, most remained unmet

S2. Main findings from all perspectives at T0 and T1

**Participation**<br>- Approximately one third had good contact with other residents<br>- Conversation partners in the nursing home were predominantly carers, who had very little time<br>- In general, residents tended to report that they had few (potential) conversation partners<br>- With respect to nursing home activity offers, there either was no demand (no matching offers/no interest) or a very strong demand	**Participation**<br>- There were different perceptions as to the extent to which social contact had to be restricted. The majority of the residents expressed that personal contact was no longer possible. One person described this as stressful<br>- The severely restricted social contact caused by the pandemic was met with understanding<br>- The majority of the residents surveyed expressed that personal contact was no longer possible<br>- In general, (greater) use was made of the telephone to maintain contact with relatives
**Dealing with dying and death**<br>- Residents deal with the last phase of life as well as dying and death differently (repression, calmly awaiting, intensive preoccupation (incl. in dreams), longing for death)<br>- Development of the idea of a "good death" from experiences with the deaths of other people (relatives and friends)<br>- Requests and expectations (most common: wanting to die alone or in company, it should be quick, composedly awaiting death)<br>- Residents essentially assume that they cannot have any influence on their last phase of life<br>- Only few specific organisational requests are defined for the last phase of life<br>- There are virtually no discussions on specific requests and expectations concerning the dying phase with relatives as well as employees<br>- In discussions about the end of life and death in general, communication with the family takes priority<br>- The possibility of hospice care is a virtual unknown, unless this has been independently experienced by relatives or friends<br>- Nursing home rituals after residents have died are known in some cases (black ribbon and book of condolence)<br>- Possibilities of saying goodbye to deceased residents are not well known<br>- Formal matters (e.g., funeral modalities) are regulated	**Dealing with dying and death**<br>- The residents surveyed overwhelmingly reported that they did not think about the last phase of life or deliberately pushed those thoughts away<br>- In terms of the organisation of the last phase of life, there was a great reliance on relatives<br>- Most respondents assumed that they would not be alone at the end of life. Above all, having trusted family members by their side was most important<br>- Two people stated that they would prefer to die alone. One described that their feelings in this respect had changed and that they would now find it unpleasant if everyone were standing around the bed at the end of their life. The other person stated that they did not want to burden the children and so had to "do it" alone

S2. Main findings from all perspectives at T0 and T1

Relatives

T0	T1
**Organisation and communication**<br>- Communication with carers predominantly took place during visits to the nursing home and only at the initiative of relatives as impromptu conversations<br>- There were no regular communication structures in a relaxed atmosphere, only the ad hoc clarification of questions/problems due to a lack of employee time as well as lack of continuity of staff<br>- No contact with other people involved in the care. Relatives had no information of the care and support provided by other professionals; the responsibilities were not clear<br>- Relatives had no “point of contact” for their concerns with respect to hospice and palliative care<br>- The needs of relatives were not taken into account by the nursing home	**Organisation and communication**<br>- Contact between residents and relatives took place by phone, video-chat, and later behind plexiglass or in the park; some relatives rated no contact as best solution in order to not confuse their residents<br>- Communication with caregivers took place by telephone<br>- Caregivers contacted relatives more often on their own initiative to provide updates. Detailed information talks (also with other professionals involved in care) did not take place, which was also not expected during the pandemic<br>- The frequency of contact depended on the employees<br>- The needs of the relatives were partly addressed by the employees<br>- Relatives tended not to address conflicts with employees. If they did, it did not lead to the desired change<br>- Dealing with conflicts was perceived as more difficult during the pandemic, because relatives were afraid that talking about unpleasant things would have a negative effect on the care relationship<br>- Relatives were very satisfied with the pandemic management in the nursing homes (situation was taken seriously, measures were consistently applied)<br>- There was a general understanding of the measures and an insight into the overall situation “It sucked, but there was nothing you could have done”
**Involvement in care and support**<br>- Relatives were most likely to contribute when it came to the interior design<br>- Their own ideas were not communicated, although the desire existed because:<br>- structures were found to be too rigid to introduce certain matters<br>- the physical condition/health of the resident was poor and relatives had no idea how they could get involved<br>- there was a fear of financial consequences<br>- There was no guidance, advice, or support by employees<br>- Opportunities for active contributions to the last phase of life were not known<br>- The involvement of relatives in the last phase of life was not addressed in advance (neither by residents nor employees)	**Involvement in care and support**<br>- Some relatives stated that their own wishes and ideas could be introduced. Others reported only little openness from the staff<br>- When fulfilling wishes, the employees tended to concentrate on one-off major events (“doing xx one last time”), the implementation of smaller, everyday wishes was not successful<br>- The implementation of wishes and autonomy attempts by the residents failed mainly due to organisational processes and a lack of willingness of employees<br>- Opportunities for guidance, advice or support by employees were not discussed during the pandemic<br>- Relatives were heavily burdened by the fact that they could not be with residents in the last phase of their life, only very little or only to a limited extent (due to protective measures)

S2. Main findings from all perspectives at T0 and T1

	- Relatives stated that they experienced the residents as heavily burdened during the pandemic-related contact restrictions. Residents who understood the pandemic situation were more likely to accept protective measures<br>- Relatives all agreed that social distancing was necessary to protect residents and visitors
**Dealing with the last phase of life**<br>- Importance of participation in care and support increased in the last phase of life. Involvement was presumed by relatives<br>- Great uncertainty existed amongst relatives in dealing with dying and death<br>- There was virtually no communication on the last phase of life with residents and employees. Reasons included:<br>- No awareness/lack of knowledge that something could be discussed<br>- Fear of addressing the topic<br>- No need (yet), as the resident was faring well<br>- Communication desired but not (no longer) possible (due to condition of the resident or lack of employee time)<br>- The possibility of integrating outpatient hospice services was largely unknown and unused<br>- Respite services offered by the nursing home were unknown, and employees did not convey external offers<br>- No detailed knowledge of the process surrounding deceased residents	**Dealing with the last phase of life**<br>- Discussions with the residents about the last phase of life did not increase, although death due to the pandemic became more present. Reasons included:<br>o The little time that remained due to limited contact options did not want to be spend with these topics<br>o The telephone was not considered to be the right medium for addressing these issues<br>- Concentration on organisational aspects (power of attorney, funeral modalities); the residents of all relatives had a power of attorney and/or care directive; no adjustments to the documents were made during the pandemic<br>- Respite services offered by the nursing home were unknown, employees did convey one external offer to one relative
